# The C-terminal protein interaction domain of the chromatin reader Yaf9 is critical for pathogenesis of *Candida albicans*

**DOI:** 10.1128/msphere.00696-23

**Published:** 2024-02-20

**Authors:** Tricia L. Lo, Qi Wang, Joshua Nickson, Bryce J. W. van Denderen, Deanna Deveson Lucas, Her Xiang Chai, Gavin J. Knott, Harshini Weerasinghe, Ana Traven

**Affiliations:** 1 Department of Biochemistry and Molecular Biology and the Infection Program, Biomedicine Discovery Institute, Monash University, Clayton, Australia; 2 Centre to Impact AMR, Monash University, Clayton, Australia; 3 Genomics and Bioinformatics Platform, Monash University, Clayton, Australia; University of Georgia, Athens, Georgia, USA

**Keywords:** *Candida albicans*, chromatin, Yaf9, YEATS domain, chromatin reader

## Abstract

Fungal infections cause a large health burden but are treated by only a handful of antifungal drug classes. Chromatin factors have emerged as possible targets for new antifungals. These targets include the reader proteins, which interact with posttranslationally modified histones to influence DNA transcription and repair. The YEATS domain is one such reader recognizing both crotonylated and acetylated histones. Here, we performed a detailed structure/function analysis of the *Candida albicans* YEATS domain reader Yaf9, a subunit of the NuA4 histone acetyltransferase and the SWR1 chromatin remodeling complex. We have previously demonstrated that the homozygous deletion mutant *yaf9Δ/Δ* displays growth defects and is avirulent in mice. Here we show that a YEATS domain mutant expected to inactivate Yaf9’s chromatin binding does not display strong phenotypes *in vitro*, nor during infection of immune cells or in a mouse systemic infection model, with only a minor virulence reduction *in vivo*. In contrast to the YEATS domain mutation, deletion of the C-terminal domain of Yaf9, a protein–protein interaction module necessary for its interactions with SWR1 and NuA4, phenocopies the null mutant. This shows that the C-terminal domain is essential for Yaf9 roles *in vitro* and *in vivo*, including *C. albicans* virulence. Our study informs on the strategies for therapeutic targeting of Yaf9, showing that approaches taken for the mammalian YEATS domains by disrupting their chromatin binding might not be effective in *C. albicans*, and provides a foundation for studying YEATS proteins in human fungal pathogens.

**IMPORTANCE:**

The scarcity of available antifungal drugs and rising resistance demand the development of therapies with new modes of action. In this context, chromatin regulation may be a target for novel antifungal therapeutics. To realize this potential, we must better understand the roles of chromatin regulators in fungal pathogens. Toward this goal, here, we studied the YEATS domain chromatin reader Yaf9 in *Candida albicans*. Yaf9 uses the YEATS domain for chromatin binding and a C-terminal domain to interact with chromatin remodeling complexes. By constructing mutants in these domains and characterizing their phenotypes, our data indicate that the Yaf9 YEATS domain might not be a suitable therapeutic drug target. Instead, the Yaf9 C-terminal domain is critical for *C. albicans* virulence. Collectively, our study informs how a class of chromatin regulators performs their cellular and pathogenesis roles in *C. albicans* and reveals strategies to inhibit them.

## INTRODUCTION

Chromatin readers control gene expression through physical interactions with posttranslationally modified histones or DNA (1). They are subunits of transcriptional and chromatin modification complexes and possess specialized domains that recognize chromatin modifications, such as acetylation or methylation. By virtue of their interaction with chromatin, readers recruit their associated complexes to DNA to execute transcriptional regulation (1).

Dysfunctional chromatin readers have been implicated in human diseases. Therefore, interest in therapeutic strategies that either disrupt or regulate their interactions with chromatin is increasing. A well-known example is that of bromo- and extra-terminal (BET) domain inhibitors, which inhibit the interactions of bromodomains with acetylated histones (2, 3). While there are challenges to their clinical application, BET inhibitors are being considered for cancer chemotherapy and other diseases, such as those driven by hyperactive immune responses (3–10).

Chromatin readers are found in species from yeast to humans. As such, they can be explored as therapeutic targets not only in human cells but also for targeting infections caused by pathogenic fungi. While we are only beginning to understand the roles of chromatin readers in fungal pathogens, studies to date already support this proposition (11). For example, the BET domain protein Bdf1 is required for cell viability of the human fungal pathogen *Candida albicans*, and its conditional inactivation reduces virulence in a pre-clinical model in mice (12). The same study reported on the discovery of inhibitors of the *C. albicans* Bdf1 bromodomains, which showed little binding to a human bromodomain and had a reasonable toxicity profile in mammalian cellular assays (12). Moreover, using a non-mammalian animal model (*Galleria mellonella* larvae), the virulence of the mold fungal pathogen *Aspergillus fumigatus* was reduced in the presence of the BET inhibitor JQ1 (13).

We have been interested in a chromatin-binding domain called YEATS (Yaf9-ENL-AF9-Taf14-Sas5). The YEATS domain recognizes crotonylated and acetylated histone lysines (14–18) and is found in several proteins that possess DNA transcription and repair functions (19). Since mammalian YEATS proteins are involved in cancerous transformations, YEATS domain inhibitors are attractive as a possible treatment of some cancers (20–27). The YEATS domain is found across fungal species (28), and the evidence for the importance of YEATS proteins in fungal virulence is building. For example, the *C. albicans* genome encodes two YEATS proteins, Taf14 and Yaf9, and homozygous inactivation of either gene reduces growth and virulence in a murine bloodstream infection model (28). Taf14 has also been shown to be required for wild-type growth and virulence of the plant fungal pathogen *Botrytis cinerea* (29). MOreover, in the human yeast pathogen *Cryptococcus neoformans,* the Yaf9-related YEATS protein, Yst1, is required for full pathogenicity in the *Galleria* model (30).

How YEATS proteins could be inhibited as an antifungal strategy remains to be understood. One approach would be to target the chromatin-binding YEATS domain, similar to the strategy taken in mammalian cells. In support of this, studies to date have shown the importance of chromatin binding in enabling the cellular roles of the fungal YEATS proteins (14, 17, 31, 32). However, mutations that inactivate chromatin binding by the YEATS domain tend to have a smaller phenotypic consequence for fungal cells than the complete absence of the YEATS protein in the null mutant, arguing that the YEATS domain is important for some but not all functions of the YEATS proteins. This has been shown in *Saccharomyces cerevisiae* with Taf14 and Yaf9 (17, 31–34), as well as for Taf14 in *C. albicans* (28) and *B. cinerea* (29). Therefore, a detailed evaluation of the functions of the YEATS proteins in fungal pathogens is needed to understand their potential as antifungal drug targets.

We previously determined that the deletion of *YAF9* in *C. albicans* strongly reduces growth and virulence in mice, even more dramatically than the deletion of *TAF14* (28). However, we did not dissect these phenotypes in detail. In this study, we show that mutations in the YEATS domain play a minor role in Yaf9’s functions in *C. albicans*. In contrast, the C-terminal domain of Yaf9 is critical for all of the functions that we tested including growth, stress responses, and virulence in the mouse bloodstream infection model. Based on our structural modeling and data for the *S. cerevisiae* homologs (34–39), the C-terminal domain of *C. albicans* Yaf9 likely mediates its interactions with Eaf2 (aka Swc4), a subunit of the histone acetyltransferase NuA4 and the chromatin remodeling complex SWR1. Collectively, our results inform on the mechanisms by which Yaf9 promotes *C. albicans* growth and virulence, and shed light on the strategy for assessing YEATS proteins as potential antifungal drug targets.

## RESULTS

### The C-terminal domain of Yaf9 is required for *C. albicans* growth, while YEATS domain mutants grow similarly to the wild-type

**Fig 1:**
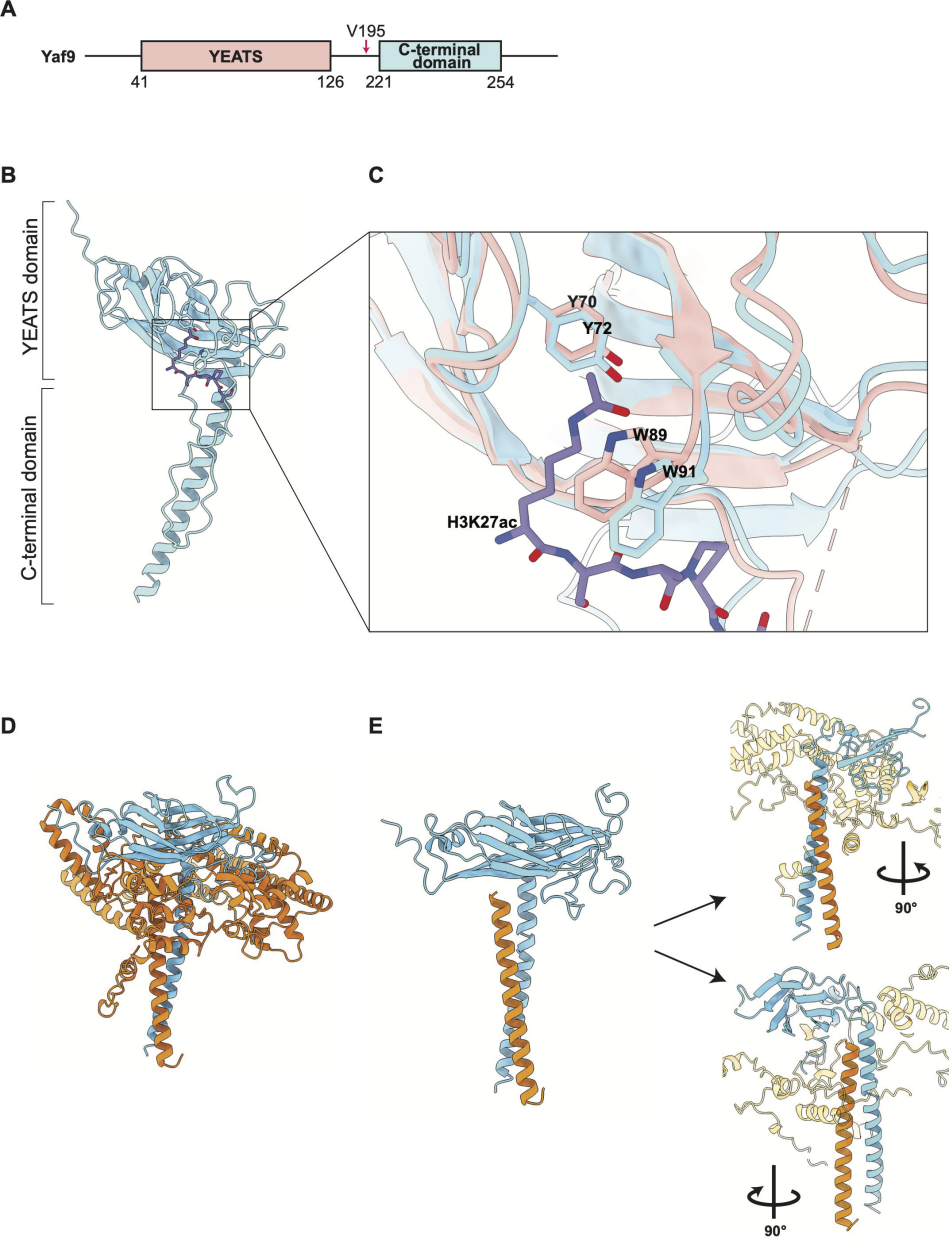
Structural models of *C. albicans* Yaf9. (A) Representation of the Yaf9 protein from *C. albicans*, with structural features indicated. V195 is the residue at which the C-terminal deletion mutant was truncated. (B) The AlphaFold predicted structure of *C. albicans* Yaf9 (AF-Q59LC9-F1_V4, cyan) superimposed with the H3K27ac (purple) from the crystal structure of *S. cerevisiae* Yaf9-YEATS domain (14) PDB ID: 6AXJ. (C) Close-up view of AlphaFold predicted structure of *C. albicans* Yaf9 (AF-Q59LC9-F1_V4, cyan) superimposed with the crystal structure *S. cerevisiae* Yaf9 (PDB ID: 6AXJ, light pink), showing amino acid residues Y72 and W91 from *C. albicans* Yaf9 (cyan) are in proximity with H3K27ac (PDB ID: 6AXJ). *C. albicans* Yaf9 Y72 and W91 correspond to Y70 and W89 in *S. cerevisiae* Yaf9. (D) AlphaFold Multimer V3 predicted structure of *C. albicans* Yaf9 (cyan) co-folded with *C. albicans* Eaf2 (orange). (E) Close-up of the predicted structural proximity of the Yaf9 ET domain helix with the Eaf2 helix.

In *S. cerevisiae* and *C. albicans,* Yaf9 is a subunit of the histone acetyltransferase NuA4 and the chromatin remodeling complex SWR1 (34–37, 39–45). In *C. albicans,* Yaf9 physically links NuA4 and SWR1 by interacting with the Eaf1 subunit *via* its YEATS domain (43). An AlphaFold model of the *C. albicans* Yaf9 protein shows the predicted structure of the N-terminal YEATS domain and the helix formed by the C-terminal domain (Fig. 1A and B). The structure of the *S. cerevisiae* Yaf9 YEATS domain has been solved, showing the importance of residues W89 and Y70 for interacting with acetylated lysine 27 in histone H3 (H3K27ac) (14). Consistent with their importance for histone interactions, these two residues are conserved in mammalian orthologs of Yaf9, YEATS2 and Gas41 (14). Our sequence alignment shows that these residues are also conserved across divergent fungal species, with complete conservation of W89 for the species we analyzed and partial conservation for Y72 (Fig. S1). The equivalent residues in the *C. albicans* Yaf9 YEATS domain are W91 and Y72.

The *C. albicans* Yaf9 structure model was superimposed onto the solved structure of the *S. cerevisiae* Yaf9 YEATS domain together with its H3K27ac ligand, showing a conserved binding pocket (Fig. 1C). Data from *S. cerevisiae* are consistent with Yaf9 using its C-terminal domain to interact with SWR1 and NuA4 (34–39). Its direct binding partner is the Eaf2 subunit (35–37). An AlphaFold Multimer prediction showed the *S. cerevisiae* Yaf9 C-terminal domain helix interacting with Eaf2 (37). Similarly, we modeled *C. albicans* Yaf9 co-folded with Eaf2 and demonstrated a possible involvement of the C-terminal domain in the interaction (Fig. 1D and E).

**Fig 2:**
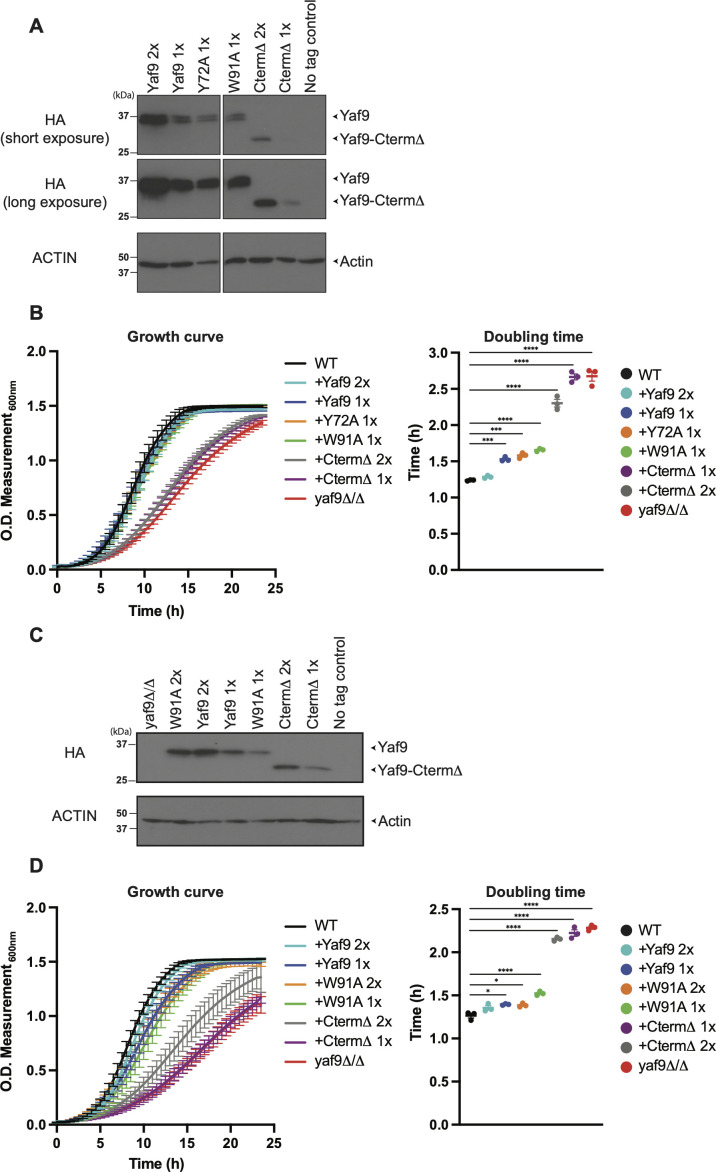
Inactivatio of the C-terminal domain of *C. albicans* Yaf9 dramatically slows growth. (**A)** Western blot of Yaf9 expression levels. Strains were grown to log phase at 30°C. The blots show two exposures; short exposure (first panel) and long exposure (second panel). The strains are *yaf9Δ/Δ* complemented with the indicated constructs (wild-type Yaf9, Yaf9W91A, Yaf9Y72A, or CtermΔ). All constructs have a C-terminal HA tag used to detect Yaf9. The copy number of Yaf9 is indicated by 1× or 2×. All samples were run on the same gel, but a lane with a construct that was not further analyzed was cropped out as indicated. The uncropped Western blot is shown in Fig. S2A. (**B)** Left panel: growth curve of the strains shown in panel (A) alongside the parental wild-type strain (SN425) and the *yaf9Δ/Δ* mutant. The strains were grown at 30°C in yeast extract peptone dextrose (YPD) + uridine media. Right panel: doubling time calculations from the growth curves. Shown are the means and SEM for three independent experiments. Statistical significance was determined using one-way ANOVA and Tukey’s multiple comparisons test (^****^P ≤ 0.0001). Only statistically significant comparisons are indicated in the figure. (**C)** Western blot as in panel (A), but with a set of independently constructed strains. Uncropped Western blots are shown in Fig. S2B. (**D)** Growth assays with the strains shown in panel (C). The growth curves and doubling time calculations were performed as in panel (B). Shown are the means and SEM for three independent experiments. Statistical significance was determined using one-way ANOVA and Tukey’s multiple comparisons test (^****^P ≤ 0.0001). Only statistically significant comparisons are indicated in the figure.

To create YEATS domain mutants, residues W91 or Y72 were mutated to alanine. Deletion of the C-terminal domain was obtained by removing residues from V195 onward (Fig. 1A). Since V195 sits in the loop that connects the YEATS domain with the C-terminal helix, the C-terminal domain deletion mutant expresses only the YEATS domain. To monitor protein expression, a 3x-HA tag was fused to the C-terminus of these constructs. The *C. albicans* strains expressing these constructs were made by integration into the genome of the homozygous deletion mutant *yaf9Δ/Δ* (see Materials and Methods). We also created a control strain by integrating a construct expressing wild-type Yaf9. Initially, we integrated one copy of these constructs into the deletion mutant and then two copies in some cases for reasons of expression levels (see below). All single-copy re-integrants expressed equally, except for the C-terminal domain deletion (CtermΔ), which was low (Fig. 2A, uncropped Western blots in Fig. S2). We, therefore, introduced two copies of the CtermΔ construct, which resulted in protein levels comparable to the one-copy re-integrants for wild-type Yaf9 or the W91A and Y72A mutants (Fig. 2A). Wild-type Yaf9 and the W91A and Y72A mutants migrated on SDS-PAGE as two bands (Fig. 2A), indicating a possible posttranslational modification.

Next, we compared the growth of these strains. We have previously shown that the *yaf9Δ/Δ* null strain is slow-growing (28), and this was reproduced here (Fig. 2B). The CtermΔ mutant also grew slowly albeit somewhat better than the *yaf9Δ/Δ* null when two copies were integrated to increase its expression levels (Fig. 2B). However, its growth was much lower than the one-copy YEATS domain mutants or wild-type Yaf9 re-integrants that showed similar expression levels on Western blots (Fig. 2A). In contrast, growth of the W91A or Y72A YEATS domain mutants was indistinguishable from the strain expressing wild-type Yaf9 (Fig. 2B, all one-copy re-integrants). All three strains grew a little more slowly than the parental wild-type strain (Fig. 2B), possibly due to a dosage effect from only one copy of the *YAF9* gene in these strains. Indeed, the introduction of another copy of wild-type *YAF9* increased its protein levels (Fig. 2A, compare first lane with two copies of Yaf9 and second lane with one copy of Yaf9) and also reverted the growth to parental wild-type levels (Fig. 2B, compare dark blue with one copy of Yaf9 with light blue with two copies of Yaf9).

In parallel, we constructed another equivalent set of strains using an independently made *yaf9Δ/Δ* null mutant. Here, we re-integrated either one or two copies of the genes encoding wild-type Yaf9, the YEATS domain W91A mutant, or the CtermΔ mutant. Western blots showed higher protein expression levels for two-copy compared to one-copy re-integrants (Fig. 2C, uncropped Western blots in Fig. S2). The expression levels of the CtermΔ mutant were comparable to W91A and wild-type Yaf9 (Fig. 2C). Growth curves for this independently constructed set of strains confirmed that the CtermΔ mutant grows very slowly, while the W91A only displays a minor growth defect (Fig. 2D). Collectively, using two sets of independently constructed mutants, we show that the C-terminal domain of Yaf9 is important for the growth of *C. albicans*, while mutations of the YEATS domain have only a small effect under these growth conditions.

### Roles of *C. albicans* Yaf9 domains in stress responses

**Fig 3:**
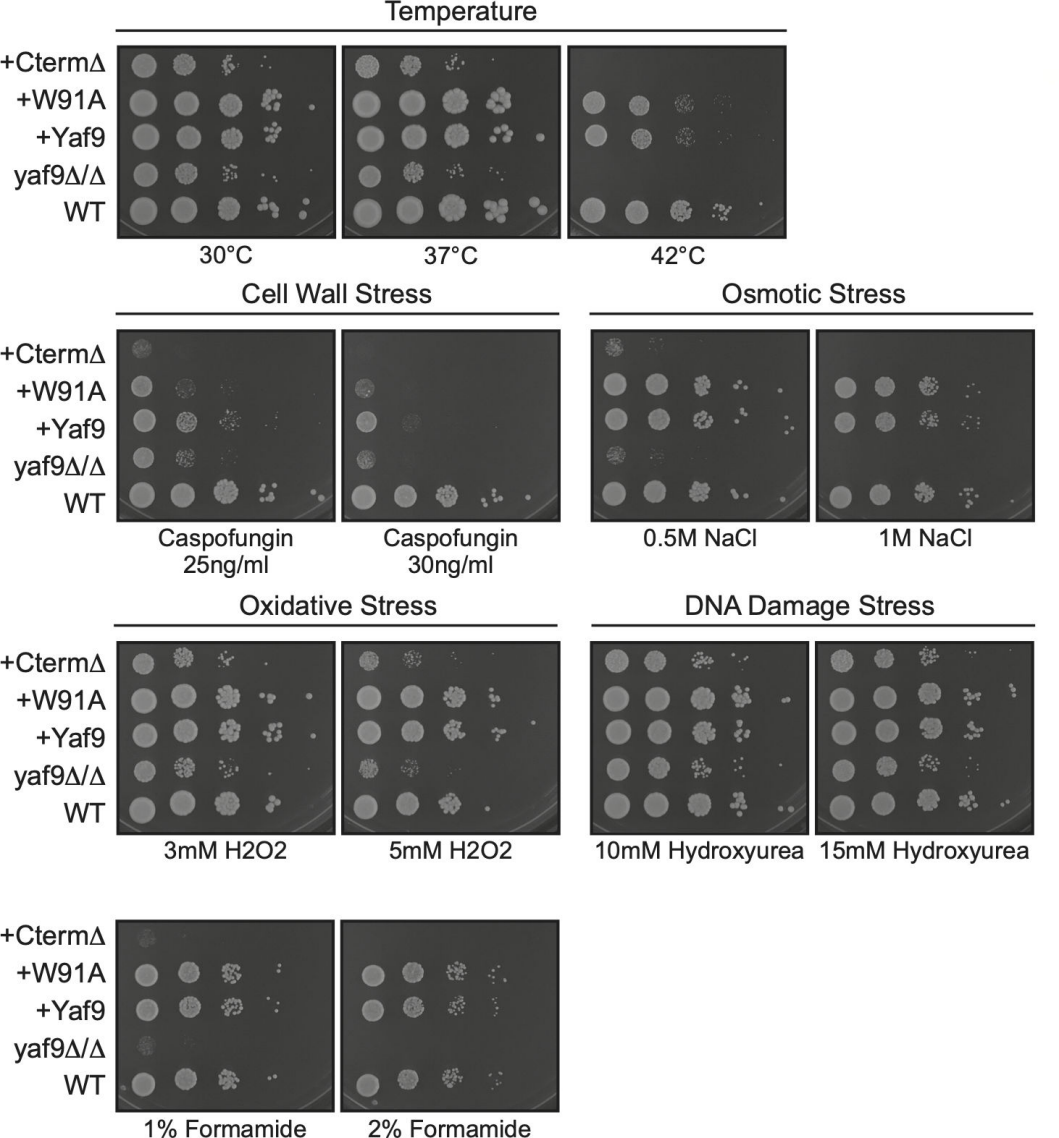
The Yaf9 C-terminal domain functions in *C. albicans* stress resistance. Ten-fold serial dilutions of fungal cultures were spotted on plates with or without the indicated compounds and incubated for 3 days before photographing. Unless indicated otherwise, the growth temperature was 30°C. The strains used here are the two-copy re-integrants shown in Fig. 2C and D.

We have previously shown that the *yaf9Δ/Δ* mutant is hypersusceptible to temperature stress (28). As a control for comparison with the domain mutants, this was reproduced here, showing that the *yaf9Δ/Δ* mutant grows slowly at 30°C and slows progressively with increasing temperatures (very slow growth at 37°C, no growth at 42°C) (Fig. 3). Complementation with wild-type Yaf9 restored growth to wild-type levels. The YEATS domain mutant W91A grew as well as the wild-type strain (Fig. 3). The CtermΔ mutant behaved like the *yaf9Δ/Δ* strain, with slow growth at 37°C and lack of growth at 42°C (Fig. 3).

In addition to temperature, the *yaf9Δ/Δ* mutant was hypersusceptible to caspofungin (cell wall stress), osmotic stress (1 M NaCl), and formamide and had a minor susceptibility to oxidative stress (H_2_O_2_) and no susceptibility to DNA damage (hydroxyurea) (Fig. 3). Complementation with wild-type Yaf9 restored growth under most of these conditions, although not completely for caspofungin particularly at higher doses (Fig. 3). The W91A showed normal (wild-type) susceptibility to most of the stressors, except a slightly higher susceptibility to caspofungin (Fig. 3). The CtermΔ mutant behaved like the *yaf9Δ/Δ* strain and was hypersensitive to formamide, NaCl, caspofungin, and H_2_O_2_ (Fig. 3). These data indicate that the C-terminal domain of Yaf9 is essential for stress responses. In contrast, mutation of the YEATS domain plays no role in most of the stress responses tested and only a minor role in cell wall stress.

### Roles of the Yaf9 domains in *C. albicans* morphology

**Fig 4:**
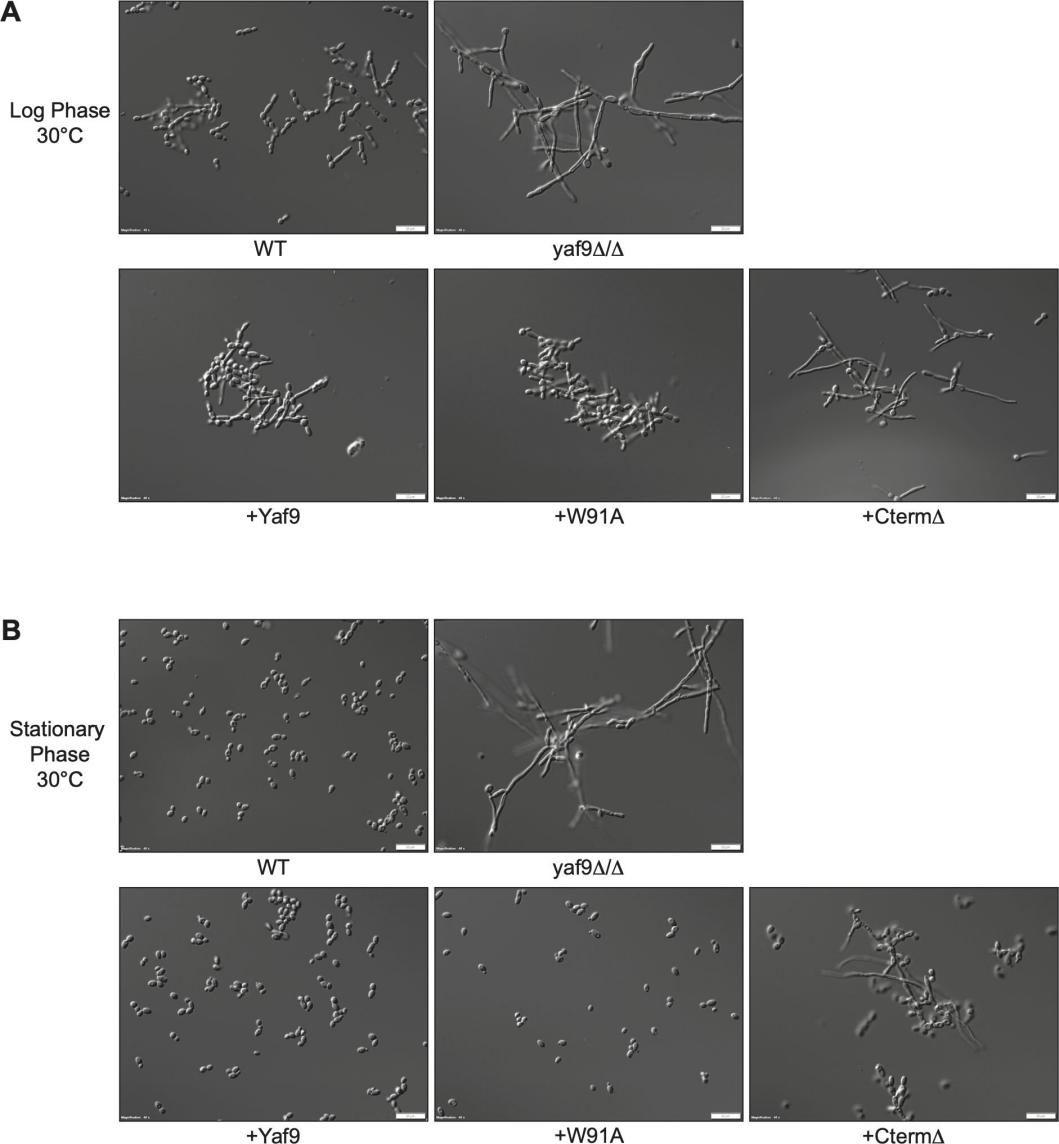
Roles of the Yaf9 YEATS and C-terminal domain C in *C. albicans* morphogenesis. (**A)** Morphology of the indicated strains in log phase growth at 30°C YPD + uridine media. Images were taken after 3 h of growth. The scale bar is 20 µm. Images are representatives of three independent experiments (see Fig. S3A). The strains used here are the two-copy re-integrants shown in Fig. 2D. (**B)** As in (A) but strains were grown to stationary phase. Images are representatives of three independent experiments (see Fig. S3B).

Wang et al. have shown that the interaction of Yaf9 with Eaf1 brings together the *C. albicans* SWR1 and NuA4 chromatin modification complexes and has important roles in determining cellular morphology (43). Specifically, the YEATS domain of Yaf9 interacts with acetylated lysine 173 of Eaf1, thus mediating the formation of the SWR1-NuA4 supercomplex during growth in yeast morphology (43). The same authors reported that the complete deletion of *YAF9* or deletion of the Yaf9 YEATS domain results in a hyperfilamentous morphology (43). Consistent with Wang et al., we found that the *yaf9Δ/Δ* mutant was hyperfilamentous and grew hyphal cells in conditions under which the wild-type strain grows predominantly as yeast (log and stationary phase, YPD, 30°C) (Fig. 4A and B; this one and two other independent experiments shown in Fig. S3). Complementation with wild-type Yaf9 or the YEATS domain mutant W91A restored a wild-type morphological appearance (Fig. 4A and B; Fig. S3). We did, however, observe more elongated cells and pseudohyphae in these complemented strains compared to the more uniform yeast morphology seen in the parental wild-type strain, particularly in log phase growth (Fig. 4A and B; Fig. S3). Examination of cell morphology across multiple fields of view in three independent experiments showed a somewhat higher propensity of the W91A mutant to display some pseudohyphal cells relative to wild-type Yaf9 (Fig. 4; Fig. S3). These data argue that, while mutation of the YEATS domain might have a small effect, it does not significantly impact cellular morphology.

In contrast, the CtermΔ mutant behaved like *yaf9Δ/Δ* with largely hyphal cells in log phase growth (Fig. 4A; Fig. S3). After prolonged growth of the cultures to the stationary phase, *yaf9Δ/Δ* and the CtermΔ mutant still showed more elongated cells, with a mixture of hyphae and pseudohyphae, but also some yeast cells (Fig. 4B; Fig. S3). Therefore, as is the case for growth rates and stress responses, the C-terminal domain of Yaf9 is essential for the proper regulation of *C. albicans* morphogenesis.

### Genomic adaptations of *C. albicans* to the loss of Yaf9

A study by the Berman lab using transposon mutagenesis in a haploid *C. albicans* strain reported that *YAF9* is an essential gene (46). As shown in our previous work (28) and here, we obtained homozygous diploid *yaf9Δ/Δ* mutants in the *C. albicans* laboratory strain SN152 [derived from the clinical isolate SC5314, which is also the parent of the Berman lab haploid strain (46–48)]. Our *yaf9Δ/Δ* mutants are slow-growing in rich medium at 30°C and grow very slowly or not at all at increased temperatures [Fig. 2 and 3 and reference (28)]. Based on these considerations, we wondered if there were genomic adaptations in our mutants that were allowing *yaf9Δ/Δ* to maintain viability. To answer this question, we sequenced the genomes of several *yaf9Δ/Δ* clones, as well as the re-integrants expressing wild-type Yaf9, the W91A mutant, and the CtermΔ mutant. In parallel, we sequenced the wild-type parental strains: the triple leucine, histidine, and arginine auxotroph SN152 used for mutant construction. SN425 is the prototrophic strain obtained by the integration of auxotrophic markers back into SN152 (48). We also sequenced the parental heterozygous deletion mutant (i.e., the strain obtained after the deletion of the first *YAF9* allele, which served as the parent for deleting the second allele).

**Fig 5:**
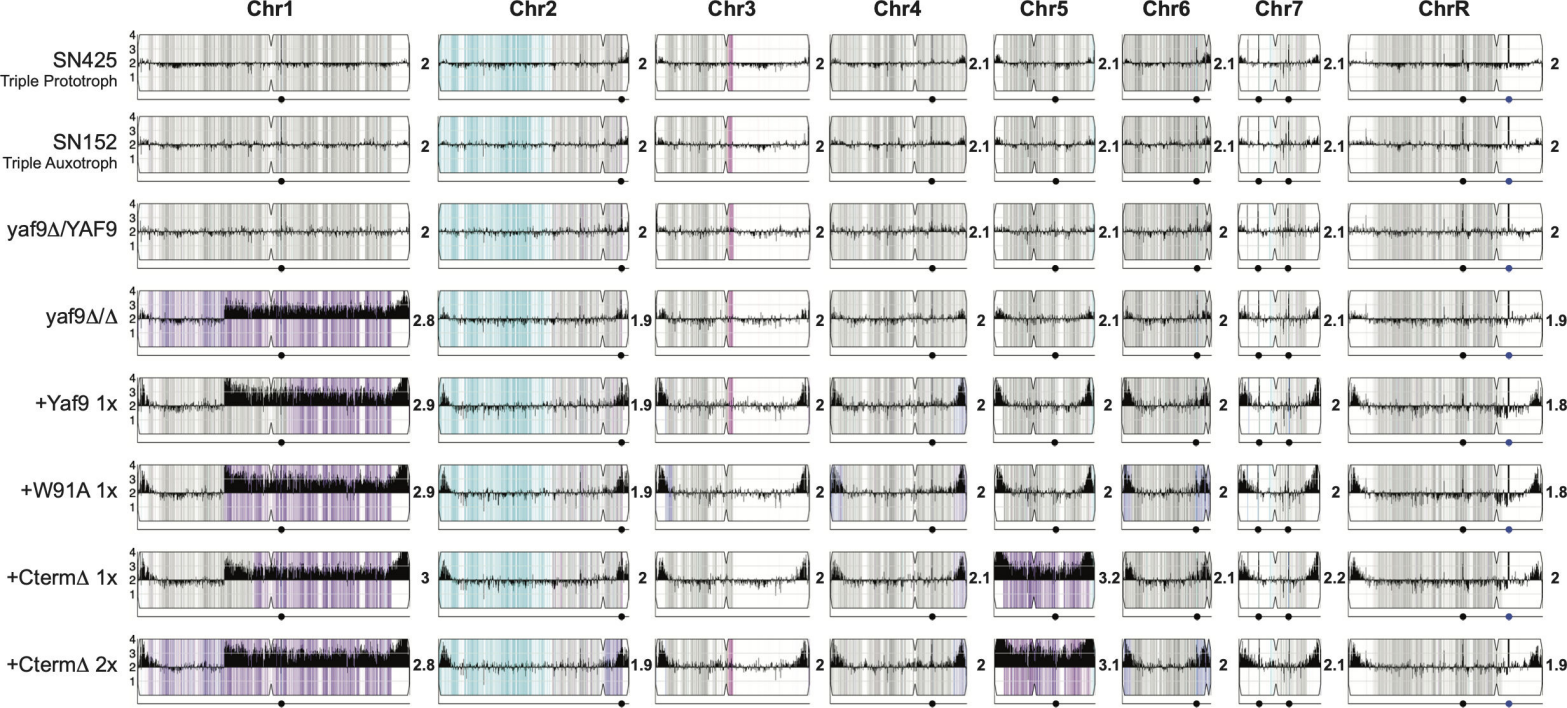
Genomic adaptation of *C. albicans* to loss of Yaf9 YMAP was used to illustrate the chromosome copy number in wild-type parentals (SN425 and SN152) and six Yaf9 derivative strains as indicated (figures generated in CNV and SNP/LOH view using http://lovelace.cs.umn.edu/Ymap/). The genomics data for SN425 is from our previous publication (51), and here, it was also annotated for heterozygosity/homozygosity. Copy number variations per position are displayed as black histograms along the length of each chromosome, and the copy number calculation is indicated next to each chromosome. The *y*-axis represents the relative chromosome copy numbers, based on the whole genome ploidy. The colors indicate heterozygosity (gray), allele “a” homozygosity (cyan), and allele “b” homozygosity (magenta). Additional independent clones are shown in Fig. S3.

Our sequencing confirmed a previous report from the Butler lab showing that SN152 contains a large loss of heterozygosity (LOH) region on chromosome 2 and also a smaller LOH region on chromosome 3 [Fig. 5 and reference (49)]. These LOH events were inherited from the strain used to make SN152 (50). We also saw the same LOH events in the prototrophic parental strain SN425 (derived from SN152) (Fig. 5; Fig. S4), sequenced in our previous study (51). The heterozygous strain *yaf9Δ/YAF9* resembled these control strains (Fig. 5; Fig. S4).

Sequencing of our *yaf9* mutant strains revealed a complex picture of genomic alterations, with chromosome aneuploidies and LOH events (Fig. 5; Fig. S4). Of the three *yaf9Δ/Δ* clones that we sequenced, two had an additional copy of chromosome 1 (segmental or full chromosome duplication), while one had an additional copy of chromosome 6 (Fig. 5; Fig. S4 ). When the wild-type *YAF9* gene was re-integrated into the two independently constructed *yaf9Δ/Δ* strains (one-copy re-integration), in one case, the additional copy of chromosome 1 was lost, and in the other, it was not (Fig. 5; Fig. S4). In contrast, both re-integrants expressing the YEATS domain mutants W91A retained chromosome 1 duplication, and so did the one strain that we sequenced, which expresses the YEATS domain mutant Y72A (Fig. S4). The CtermΔ mutants retained chromosome 1 duplication and had an additional duplication of chromosome 5, which was seen in four independent clones (Fig. 5; Fig. S4 ). These data suggest that the essential or almost essential function of the *YAF9* gene is compensated during mutant construction by aneuploidies of chromosome 1 or 6, and additionally, a selection for extra chromosome 5 is suggested for the *C. albicans* strains that lack the Yaf9 C-terminal domain.

### Roles of the Yaf9 domains in *C. albicans* immune interactions and virulence

To understand the roles of Yaf9 during infection, we used two models: primary mouse macrophages (bone marrow-derived or “BMDMs”) and the murine tail vein injection model of bloodstream disseminated disease. The large proportions of hyperfilamentous morphologies in cultures of *yaf9Δ/Δ* and CtermΔ mutants presented challenges for phagocytosis by macrophages, as well as during tail vein infection of mice. To circumvent this, we used saturated overnight cultures of these mutants that contained a higher proportion of yeast and shorter pseudohyphae (see Fig. 4) and filtered them to remove the highly hyphal cells (see Materials and Methods). We realize that filtering out of the filamentous cells could induce some cell population bias, but it was the only way to ensure that we could effectively challenge macrophages and infect mice with these strains.

**Fig 6:**
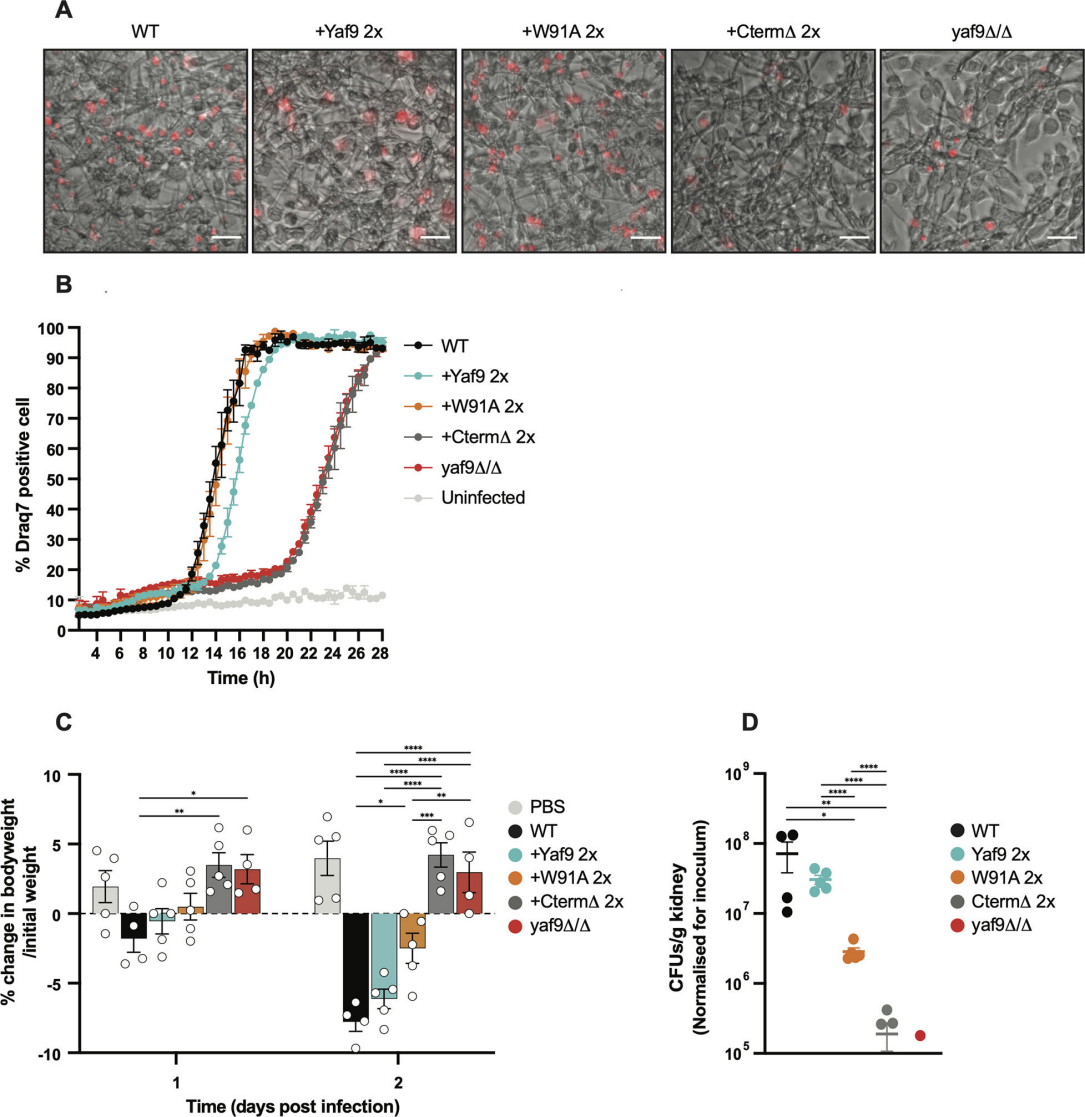
The C-terminal domain is essential for Yaf9 virulence in cellular and animal infection models. (**A)** Microscopy images of BMDMs infected with the parental wild-type strain (SN425), *yaf9Δ/Δ,* and the *yaf9Δ/Δ* re-integrants with either wild-type Yaf9, W91A, or CtermΔ constructs (two-copy-re-integrants). The multiplicity of infection (MOI) was 3 *Candida*:1 macrophage, and the images shown were taken 16 h after infection. They are overlaid with membrane-impermeable DRAQ7 dye (red) to indicate macrophages that have been permeabilized (as an indicator of cell death). The scale bar is 100 µm. (**B)** Graphical quantification for the experiments in (A). The cumulative DRAQ7-positive macrophages were counted over 24 h. Shown are the mean and SEM for two independent experiments. At least 2,000 macrophages were imaged in each experiment for each of the experimental groups. (**C)** Percentage weight change of mice infected with the indicated strains [same as in panel (A)]. The “uninfected” mice were mock-infected with sterile 1× PBS. Each data point is from an individual animal. A two-way ANOVA statistical analysis was performed followed by Tukey’s multiple comparisons test with ^*^P < 0.05, ^**^P < 0.01, ^***^P < 0.001, and ^****^P < 0.0001. Shown are the statistical comparisons for all strains with the parental WT, and the comparison between the Yaf9 vs W91A re-integrant and the *yaf9Δ/Δ* null strain compared to the CtermΔ. All comparisons are shown in Dataset S1. (**D)***C. albicans* colony-forming units from kidneys at day 2 postinfection from the experiments in (C). Each data point is from an individual animal. CFUs were normalized to the infection inoculum. Shown are the mean and SEM. A two-way ANOVA statistical analysis was performed followed by Tukey’s multiple comparisons test with ^*^P < 0.05 and ^**^P < 0.01. Shown are the statistical comparisons for all strains with the parental WT and the comparison between the Yaf9 vs W91A re-integrant and the *yaf9Δ/Δ* null strain compared to the CtermΔ. All comparisons are shown in Dataset S1.

*In vitro*-cultured primary BMDMs were incubated with our *yaf9* mutant strains. Following phagocytosis, the macrophages were assessed for viability by live cell microscopy for 24 h as previously described (52). The W91A mutant behaved similarly to the wild type: it made hyphae, escaped, and caused macrophage cell death with similar kinetics (Fig. 6A and B). In contrast, the *yaf9Δ/Δ* and CtermΔ mutants showed a large delay of around 8 h in triggering appreciable macrophage cell death (Fig. 6A and B). We have previously reported that *yaf9Δ/Δ* is avirulent in mice (28). This was reproduced here as a control: *yaf9Δ/Δ* was avirulent as judged by the lack of weight loss in the infected animals at the experimental endpoint (48 h) and lack of proliferation in infected kidneys (Fig. 6C and D).We further show here that, consistent with the low virulence of the *yaf9Δ/Δ* mutant, the infiltration of immune cells into infected kidneys (neutrophils, monocytes, and monocyte-derived dendritic cells) was dramatically reduced in *yaf9Δ/Δ*-infected mice compared to wild type-infected mice (Fig. S5A and B). Also consistent with low virulence, the numbers of structural kidney cells were not reduced in *yaf9Δ/Δ*-infected mice, unlike wild type-infected mice where their reduction indicated tissue damage (Fig. S4A and S5B). The CtermΔ mutant behaved similarly to *yaf9Δ/Δ*, in that there was no significant change in body weight compared to phosphate-buferred saline (PBS)-injected control animals, whereas mice infected with the wild type, the complemented strain (Yaf9-2X), and the W91A mutant lost ~3%–8% of weight (Fig. 6C). Further, we observed very low proliferation of the CtermΔ strain in the kidneys of these mice (Fig. 6D). It should also be noted that both the *yaf9Δ/Δ* and CtermΔ mutants displayed a 30%–40% drop in viability *in vitro* even as we attempted to adjust the inoculum to be equal to the wild type (Dataset S1). We, therefore, normalized the CFUs to the inoculum.

The W91A mutant was virulent, although the weight loss was a little lower in mice infected with the W91A mutant relative to its control strain Yaf9-2X (Fig. 6C). The W91A strain also displayed a small reduction in the CFU count in kidneys (Fig. 6D). A trend toward lower kidney CFUs for the W91A strain was also seen in another independent experiment (Fig. S5C and D). Collectively, these data show that the mutation of the Yaf9 YEATS domain results in a modest loss of virulence in mice, while loss of the C-terminal domain renders *C. albicans* avirulent.

## DISCUSSION

Chromatin reader domains, such as bromo or YEATS, are promising therapeutic targets for cancer (4). Recent work has suggested that these domains could also be targeted for infectious diseases caused by fungi (11, 12). For this to be a viable strategy, inactivation of the reader domain should lead to loss of viability and/or virulence of the fungal pathogens. In this report, we show data that indicates that targeting the Yaf9 YEATS domain is unlikely to be a credible antifungal strategy for *C. albicans*. This conclusion is based on the fact that the mutation of two highly conserved residues that are important for histone binding by the Yaf9 YEATS domain did not lead to any strong phenotypes in *C. albicans*. The Yaf9 W91A mutant showed at best a very minor growth retardation and mostly normal stress responses, with only modestly increased susceptibility to cell wall stress (caspofungin) and minor reduced virulence in the murine systemic infection model. Similarly, the Yaf9 Y72A mutant grew normally. Notably, in order to reveal a possible phenotype, we tested the W91A mutant in two scenarios where *C. albicans* encounters significant stressors and nutrient deprivation: infected macrophage and proliferation *in vivo* in mouse kidneys. However, even in these extreme conditions, the W91A mutant escaped and killed macrophages normally, and it only showed a modest reduction in proliferative capacity in mouse kidneys.

Work in *Saccharomyces cerevisiae* supports our conclusion that the mutation of the Yaf9 YEATS domain leads to mild phenotypes. A recent study showed that the chromatin-binding activity of Yaf9 is important for transcriptional and metabolic regulation during the yeast metabolic cycle (YMC) (32). However, consistent with our findings, inactivation of the *S. cerevisiae* Yaf9 YEATS domain by the W89A mutation (which corresponds to W91A in our *C. albicans* mutant, see Fig. 1) only resulted in a modest delay in the YMC, unlike the *yaf9Δ* null strain for which the metabolic cycle was disrupted (32). Moreover, the W89A mutant showed milder stress phenotypes than the *yaf9Δ* null, with the only strong susceptibility being to DNA-damaging agents (32). Another study that used different mutations in the *S. cerevisiae* Yaf9 YEATS domain came to the same conclusions (34). However, in contrast to our data in *C. albicans* (Fig. 3), the *S. cerevisiae* Yaf9 mutants W89A and Y70A both showed hypersusceptibility to formamide (14). At present, we do not have an explanation for this difference. Although we could not find strong phenotypes for the YEATS domain mutants, it is possible that the chromatin-binding activity by the Yaf9 YEATS domain regulates *C. albicans* biology under specific conditions that we have not tested. Based on *S. cerevisiae* data, this would most likely be DNA damage (32). We note that we have only tested hydroxyurea (no susceptibility), but other types of damaging agents might reveal a role.

Our data shows that the C-terminal domain of Yaf9 is critical to its functions. This assertion is based on the fact that the CtermΔ mutant phenocopied the *yaf9Δ/Δ* null strain for all of the phenotypes that we tested, including growth reduction, stress hypersusceptibility, and loss of virulence. Data in *S. cerevisiae* support our conclusions on the key roles of the Yaf9 C-terminal domain in stress responses (34). Our genomics analyses further support a critical role for the Yaf9 C-terminal domain in *C. albicans*. The homozygous deletion mutant yaf9Δ/Δ had chromosomal aneuploidies (predominantly chromosome 1), presumably to compensate for loss of essential functions. The CtermΔ mutant had an additional aneuploidy of chromosome 5, which was found in four independently constructed clones (Fig. 5; Fig. S4). This suggests that the expression of the Yaf9-YEATS domain alone (as is the case in the CtermΔ mutant) has a dominant negative effect, which it compensates for by chromosome 5 aneuploidy. Importantly, although these aneuploidies are likely to have enabled the *yaf9Δ/Δ* and the CtermΔ mutants to grow, they did not rescue virulence as both mutants were completely avirulent in the mouse model and essentially unable to proliferate in kidneys.

The structural prediction by AlphaFold models the *C. albicans* Yaf9 C-terminal domain as a helix (Fig. 1). This helix is amphipathic, consistent with it being a protein–protein interaction domain. Experimental and modeling data of *S. cerevisiae* Yaf9 interactions with NuA4 and SWR1 and specifically the Eaf2 subunit show involvement of the C-terminal domains of both proteins (34–38). Similarly, our modeling of the *C. albicans* Yaf9-Eaf2 interaction supports a requirement for the C-terminal domain (Fig. 1D and E). Based on these considerations, we propose that Yaf9’s role in *C. albicans* critically depends on its ability to use its C-terminal domain to interact with Eaf2 and thereby incorporate within SWR1 and NuA4.

A detailed structural and functional understanding of *C. albicans* Yaf9 roles within SWR1 and NuA4 is still to be obtained, but data from *S. cerevisiae* offer insights into why losing Yaf9 from SWR1 or NuA4 might be detrimental to cell function. The deletion of *S. cerevisiae YAF9* does not compromise NuA4 complex composition or global levels of histone H4 acetylation, although acetylation at specific genomic positions (telomeres) is reduced (34, 38, 42). The more dramatic role for *S. cerevisiae* Yaf9 is within SWR1, where the deletion of *YAF9* results in a significant loss of several subunits (Bdf1, Eaf2/Swc4, Arp4, and Act1) and reduces the deposition of the histone variant H2A.Z (yeast Htz1) by SWR1 (34, 38, 44, 53). Given the importance of the C-terminal domain of Yaf9 for its interaction with Eaf2 and the fact that Eaf2 is encoded by an essential gene in both *S. cerevisiae* and *C. albicans* (38, 46), we propose that the loss of Eaf2 (and possibly other subunits of SWR1) explains the strong phenotypes that we observed with the *C. albicans* mutant lacking the Yaf9 C-terminal domain.

The results that we show here for Yaf9 parallel what we have previously shown for the other *C. albicans* YEATS domain reader, Taf14. Specifically, a mutant in which the chromatin-binding activity of the Taf14 YEATS domain was inactivated grew normally in lab conditions and mouse kidneys, but inactivating the Taf14 C-terminal domain caused reduced growth and virulence similar to the *taf14Δ/Δ* null strain (28). It is interesting that in *B. cinerea*, the Taf14 YEATS domain mutant showed reduced ability to cause lesions following the inoculation of plants (29), suggesting that the importance of the YEATS domain for virulence may vary between fungal species. However, consistent with our work in *C. albicans*, the C-terminal domain is critical for *B. cinerea* Taf14 functions (29).

In summary, our study sheds new light on chromatin regulation as a possible therapeutic target in fungal infections by exploring the potential of the YEATS domain readers, which are being explored for inhibitor development for cancer treatments (20–27). Our studies with *C. albicans* Yaf9 (this study) and Taf14 (28) argue that, in contrast to cancer cells, the inhibition of the YEATS domain would not be efficacious against this pathogen. This also contrasts with the antifungal potential of inhibitors that target chromatin binding by bromodomains (11, 12). Our data argue that a more beneficial therapeutic strategy against fungal YEATS proteins would be to target the C-terminal domain, as this domain is universally essential for YEATS protein functions across fungi in studies to date, and its inactivation dramatically reduced both growth and virulence *in vivo*. The Yaf9 and Taf14 C-terminal domains are protein–protein interaction modules. Although this is challenging for therapeutic application, in principle, it is possible to target protein–protein interaction domains to disrupt function, a strategy so far predominantly explored in the cancer field (54, 55). Whether and how these sorts of strategies could be adopted for pathogenic fungi remain to be seen.

## MATERIALS AND METHODS

### Strain construction and culture conditions

The *C. albicans* strains used in this study are listed in Table S1. The *yaf9* mutant strains were generated in the SN152 strain (with *his1*^−^ *leu2*^−^ *arg4*^−^ auxotrophies). As outlined in our previous study (28), stepwise deletion of the *YAF9* alleles was done using the *Candida dubliniensis HIS1* and *Candida maltose LEU2* markers to create a homozygous *yaf9Δ/Δ* deletion strain (*HIS1*^+^ *LEU2*^+^ *arg4*^−^), and the *C. dubliniensis ARG4* was inserted into the *LEU2* locus of the mutants to produce a fully prototrophic strain (47, 48). A *yaf9* mutant strain with a C-terminal domain deletion, wild-type Yaf9-complemented strain, and complementation strains with point mutants in the YEATS domain (W91A and Y72A) were generated in both single- and double-copy formats by re-integration into the *yaf9Δ/Δ* mutant genome. Single-copy re-integrants were created by introducing one copy of the constructs at the *LEU2* locus using the *C. dubliniensis ARG4* marker for selection and are delineated as “1×” in the text and figures. The double-copy re-integrants were created by introducing a second copy into the respective 1× strain at the endogenous *YAF9* locus upstream of the *yaf9∆::HIS1* using the *NAT1* selectable marker. These strains are delineated as “2×.” The expression of all Yaf9 constructs is driven by their endogenous promoter and terminator regions. All allelic variation of Yaf9 was generated by PCR from GeneScript synthesized plasmids with the following IDs: for C-terminal deletion A82258, for Yaf9 full-length complement A86507, for *yaf9*W91A allele A83888, and for *yaf9*Y72A allele A82262. All strains were genotyped by PCR (with primers found in Table S2) and/or sequencing to confirm respective sequence changes.

All strains were maintained on YPD media (constituting 1% yeast extract, 2% peptone, 2% glucose, and 80 mg/mL uridine, with the addition of 2% agar). For overnight liquid cultures required for Western blots, growth curve assays, yeast plate growth tests, or cell morphology, a single colony of the respective *C. albicans* strains from a freshly streaked YPD plate was inoculated into a liquid YPD medium and grown for 18 h at 30°C. For yeast plate growth tests on various temperatures or stressors, 10-fold serial dilutions of yeast cultures spotted on YPD media were supplemented with 2% of glucose. All images were taken after 3 days of incubation at 30°C.

### Isolation of murine bone marrow-derived macrophages

Experiments involving mice for BMDM isolations were approved by the Monash University animal ethics committee under approval number ERM25488. BMDM isolation and differentiation were performed as previously described (52). Briefly, to prepare BMDMs, the tibias and femurs were harvested from 6–8-week-old C57BL/6 mice [obtained from the Monash Animal Research Platform (MARP)], and the bone marrow was flushed using media (RPMI 1640 supplemented with 12.5 mM HEPES, 10% fetal bovine serum (FBS), 20% L-cell conditioned medium, and 100 U/mL of penicillin–streptomycin) to release the monocytes. Cells grown on standard plastic Petri dishes were allowed to differentiate into BMDMs for 5–7 days before use in live cell imaging experiments.

### Live cell imaging of *C. albicans*-infected macrophages

Live cell imaging experiments were performed as described previously (52). Briefly, differentiated macrophages were gently scraped from Petri dishes using a cell scraper (BD Falcon), seeded in 96-well tissue culture-treated plates at 1 × 10^5^ cells/well, and then incubated overnight at 37°C with 5% CO_2_. The next day, macrophages were stained with 1 mM CellTracker Green CFMDA dye (Thermo Fisher C7025) for 35 min in serum-free RPMI 1640 (12.5 mM HEPES, 20% L-cell conditioned medium, and 100 U/mL of penicillin–streptomycin) and were then infected with wild type (SN425), *yaf9*∆/∆ + *YAF9* (2×), *yaf9*∆/∆ + *yaf9* W91A (2×), *yaf9*∆/∆ + *yaf9* Cterm∆ (2×), or *yaf*9Δ/Δ strains at a MOI of 3:1 (yeast to macrophages) in a BMDM medium. Cultures from the *yaf9*∆/∆ and *yaf9*∆/∆ + *yaf9* Cterm∆ (2×) strains were filtered through a 10-µm nylon filter (Millipore) before re-suspension to remove the long hyphal filaments in these strains that would preclude phagocytosis by macrophages. Co-incubation of *C. albicans* with macrophages was done for 1 h, followed by washing with PBS and then replacing with BMDM media containing 0.6 mM DRAQ7 (Abcam) for tracking macrophage membrane permeabilization. Imaging was performed at 30-min intervals over 24 h on a Leica DMi8 Live Cell Imaging System and HC PL FLUOTAR L with a 20×/0.40 Dry PH1 CORR objective. The imaging data were analyzed and quantified using CellProfiler 2.1.172 (56) as previously described (57) and plotted using Prism 9.0 (GraphPad Software, San Diego, CA, USA) software.

### Western blot analysis

Overnight stationary phase cultures of Yaf9 strains were diluted to an OD_600 nm_ of 0.2 in 10 mL YPD media with uridine and grown at 30°C to log phase for 3 h before harvesting for protein extraction after which pelleted tissue was frozen in dry ice immediately and stored at −80°C. Proteins were extracted as described previously (28). Prior to running gels, samples were boiled at 100°C for 5 min and then were loaded on a 12% SDS-PAGE and transferred to a PVDF membrane. Membranes were blocked with 5% milk in tris-buffered saline with Tween20 (TBS-T) for 1 h and then incubated with gentle shaking with diluted primary antibodies, either anti-actin antibody (Sigma MAB1501) as a control or anti-HA, 11 Epitope Tag Antibody (BioLegend 901513) at a 1:5,000 (actin) or 1:2,000 (HA) dilution in blocking buffer for 1 h at room temperature. Membranes were then washed three times for 10 min each with TBS-T. A horseradish peroxidase (HRP)-conjugated anti-mouse IgG secondary antibody (Sigma A4416) was used at a 1:10,000 dilution in 5% milk powder in TBS-T, incubated for 1 h at room temperature, and washed three times for 10 min each in TBS-T. A Clarity Western ECL substrate (SuperSignal West Dura Extended Duration Substrate; Thermo Fisher 34075) was used to detect the actin and Yaf9 protein, by exposure to a FUJI medical X-ray film. Actin was used as the loading control. The membrane was stripped by washing in 0.5 M sodium hydroxide for 10 min and washed with water, TBS-T, and water for 5 min each. After this, the membrane was dried and then used for the detection of the levels of actin.

### Growth curves

For growth curve assays, *C. albicans* strains were cultured as described under “Strain Construction and Culture Conditions”. Assays were run in YPD media supplemented with 2% of glucose. Assays were conducted in a 96-well plate in 200 µL. Growth was determined by optical density (OD) measurements at 600 nm using a Tecan Spark 10M plate reader at 1-h intervals over a 24-h period. Doubling times for strains used were calculated using the Growthcurver package in Rstudio (https://cran.rproject.org/web/ packages/growthcurver/vignettes/Growthcurvervignette.html#output-metrics).

### Microscopy

Images in Fig. 4; Fig. S3 were taken on the Olympus IX81 Imaging System. For stationary phase culture images, strains were grown overnight for 18 h, washed with PBS, and re-suspended before imaging. For log phase culture, strains were grown overnight for 18 h, diluted to OD_600 nm_ of 0.2, and grown for a further 3 h in a YPD medium supplemented with 2% glucose at 30°C with shaking at 200 rpm. After 3 h, cells were harvested and vigorously vortexed. All harvested cells were centrifuged at 3,000 rpm, washed with PBS, and re-suspended before imaging. For mounting on slides, 3 µL of culture was mixed with an equal volume of mounting medium (3 µL) on microscopy slides, sealed with coverslips, and visualized by microscopy (40× magnification).

### Whole genome sequencing

Genome sequencing was performed on the wild type (YCAT641, SN425), wild-type (YCAT638, SN152) *YAF9*/*yaf9∆* (two independent clones) (YCAT929 and 930) and *yaf9Δ/Δ* clone (three independent clones) (YCAT936, 948, and 949), *yaf9∆/∆ + YAF9* 1× (two independent clones) (YCAT1142 and 1248), *yaf9∆/∆ + yaf9* W91A 1× (two independent clones) (YCAT1204 and 1254), *yaf9∆/∆ + yaf9*Y72A 1× (YCAT1257), *yaf9*∆/∆ + *yaf9* Cterm∆ 1× (two independent clones) (YCAT1201 and 1251), and *yaf9*∆/∆ + *yaf9* Cterm∆ 2× (four independent clones) (YCAT1212, 1282, 1283, and 1284) strains (Table S1). Strains were grown for 18 h in a YPD medium at 30°C, and genomic DNA was extracted via the standard phenol/chloroform/isoamyl alcohol (25:24:1) extraction method. Whole genome sequencing (WGS) was performed by Charles River microbial solutions on a MiSeq V3 machine as a paired-end sequencing run. WGS libraries were prepared with DNA Illumina Prep Library preparation with unique dual indexing, using an insert size of 300 bp. To map chromosome copy number changes and genomic variations, the sequenced data were uploaded to the YMAP analysis site (58) and mapped against the SC5314 reference genome (ver. A21-s02-m08-r09) (58). The results of this analysis are shown in Fig. S4. Genome sequence data have been deposited in Sequence Read Archive (SRA) under accession number PRJNA1035255. The SN425 strain genomic data are from our previous study (51) and are deposited under Bioproject ID PRJNA885583.

### Animal infection

Female BALB/c mice aged 7–9 weeks old (17–23 g) were obtained from the MARP under the approval of the Animal Ethics Committee at Monash University (ERM #25855). Mice were housed in filter-topped cages and fed autoclaved food and water under a 12-h light–dark cycle. Following 5–7 days of acclimatization, mice were systemically infected by injection into the tail vein with 3 × 10^5^ CFUs of wild-type (YCAT641, SN425), *yaf9∆/∆ + YAF9* (2×), yaf9∆/∆ + *yaf9* W91A (2×), *yaf9*∆/∆ + *yaf9* Cterm∆ (2×), or *yaf9∆/∆* strains. Infection strains were grown in a liquid YPD medium in a shaking incubator at 30°C for 18 h, washed twice in sterile PBS, and re-suspended in PBS at a concentration of 3 × 10^6^ cells/mL. The *yaf9*∆/∆ + *yaf9* Cterm∆ (2×) and *yaf9∆/∆* strain cultures were filtered through a 10-µm nylon filter (Millipore) before re-suspension to remove long hyphal filaments specific to these strains. For each tested strain, mice were infected in groups of five with an additional naïve PBS-only injection control. Mice were monitored and weighed twice daily followed by humane euthanasia by CO_2_ inhalation when they reached the experimental end point (48 or 72 h) as per our approved protocol. Kidney fungal load was determined as previously described (52). Homogenates were serially diluted in sterile PBS and plated on YPD + uridine media and incubated at 30°C for 3 days before CFUs were counted to calculate fungal burden (CFUs/g of kidney).

### Immune cell enumeration from infected kidneys

Mice were euthanized, and the left kidneys were harvested. Kidneys were lacerated using surgical scissors and suspended in RPMI 1640 (Sigma), containing DNaseI (0.25 mg/mL; Roche) and type IV collagenase (1.75 mg/mL; Worthington). Samples were agitated continuously (RT, 40 min), followed by the addition of 0.01 M EDTA and further agitation (5 min). The single-cell suspension was passed through a 70-µM nylon mesh filter (Macs Miltenyi) and treated with red-cell lysis buffer (RCRB; ammonium chloride; home-made solution). Residual RCRB was removed, and tubular debris was allowed to sediment (30–60 s) before transferring the suspension to a fresh tube. Samples were then re-suspended in exactly 2 mL of 0.01 M EDTA bss + 2% FCS (FACS buffer) and 1/20th transferred to a 96-well U-bottom plate (Falcon). Fc receptors were blocked using purified rat gamma globulin (0.5 mg·mL, RatIg) + mouse FC block (used per manufacturer’s instruction; BD) in FACS buffer (10 min), before staining with a 2× fluorochrome-conjugated antibody cocktail (30 min, on ice). Residual antibody was removed, and samples were re-suspended in FACS buffer containing propidium iodide for dead cell exclusion (0.5 µg/mL). Analysis was performed using the Aurora spectral flow cytometer, unmixing with Spectroflo software (Cytek Biosciences), and gating with FlowJo v10.9.0 (BD Life Sciences). Data were gated using an adapted strategy from reference (59). Relevant cell populations were defined as CD45^−^ (kidney structural cells) or CD45^+^ (hematopoietic cells). Further investigation of the CD45^+^ compartment was delineated using the following antigens: neutrophils (Ly6G^+^), infiltrating monocytes (Ly6C^+^), kidney resident macrophages (F4/80^int^; CD11b^int^; CD64^+^, MHCII^+^), and monocyte-derived dendritic cells (Ly6C^+^; MHCII^+^; CD11c^+^). Cells were enumerated using the volumetric measurement of the Aurora cytometer, final resuspension volume, and the proportion of organs taken for flow cytometry. Antibody dilutions were as follows: CD45 (1:200), F4/80 (1:100), CD11b (1:300), CD11c (1:200), MHCII (1:300), LyC6 (1:200), Ly6G (1:200), and CD64 (1:100).

### Protein structure prediction and multiple sequence alignment for Yaf9

The structure of Yaf9 from *C. albicans* was predicted using AlphaFold2 (60). The amino acid sequence of Yaf9 C7_03770C_A (Candida genome database, Candida Genome Database_Yaf9) was folded in ColabFold v1.5.3 using the default parameters (mmseq2_uniref_env and unpaired_paired). Five models were generated, and the model with the highest confidence (pLDDT = 83.4, pTM = 0.769) was used for downstream structural analysis. The predicted Yaf9 structure was superimposed with the crystal structure of *S. cerevisiae* Yaf9-YEATS domain (PDB ID: 6AXJ) using Matchmaker in UCSF ChimeraX (61). The co-complex prediction of Yaf9 C7_03770C_A (Candida Genome Database_Yaf9) and Eaf2/Swc4 CR_00400C_A (Candida Genome Database_Eaf2/Swc4) was carried out using AlphaFold2 Multimer (62). Five models were generated, and the model with the highest confidence in both the ET domain and Eaf2 helix was used for downstream structural analysis in UCSF ChimeraX.

MUSCLE (https://www.ebi.ac.uk/Tools/msa/muscle/) was employed to generate multiple sequence alignments of the Yaf9 YEATS domain sequences from the fungal species indicated in Fig. S1.

### Statistical analysis

All statistical analyses in this study were conducted in GraphPad Prism 9.0 (GraphPad Software, San Diego, CA, USA). One- or two-way ANOVA with Tukey’s multiple comparisons tests was conducted on doubling time, kidney fungal burden CFU counts, and animal weight loss experiments. Student’s *t*-test with Tukey’s multiple comparisons test was conducted on the kidney fungal burden CFU counts. For all experiments, a *P*-value of less than 0.05 was considered as statistically significant. Data to construct graphs in the figures are provided in Dataset S1.

## SUPPLEMENTAL MATERIAL

Data Set S1 msphere.00696-23-s0001.xlsx Numerical data for the figures.

Supplemental figures msphere.00696-23-s0002.pdf Figures S1-S5.

Table S1 msphere.00696-23-s0003.pdf Reagents and strains used in the study.

Table S2 msphere.00696-23-s0004.pdf Primers used in the study.

ASM does not own the copyrights to Supplemental Material that may be linked to, or accessed through, an article. The authors have granted ASM a non-exclusive, world-wide license to publish the Supplemental Material files. Please contact the corresponding author directly for reuse.
