# Supplementary material for: The C-terminal protein interaction domain of the chromatin reader Yaf9 is critical for pathogenesis of Candida albicans
Source: mSphere. 2024 Feb 20;9(3):e00696-23. doi: 10.1128/msphere.00696-23 (PMC10964406; doi:10.1128/msphere.00696-23)
Supplement: Supplemental figures — Figures S1-S5. [file msphere.00696-23-s0002.pdf]

Figure S1

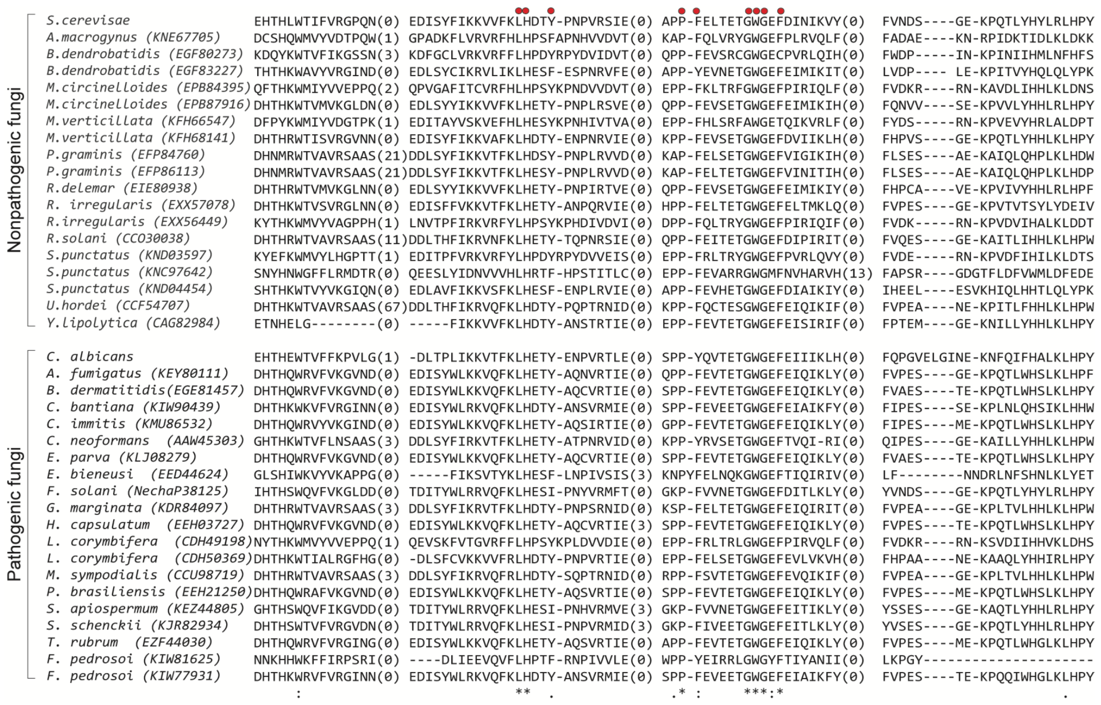

Figure S1. Sequence alignment of the Yaf9 YEATS domain across fungal species

The numbers in brackets represent the number of omitted amino acids. Amino acids important for YEATS domain structure and function are highlighted using red dots. Identical residues: “*”; strongly similar residues: “:”; weakly similar residues: “.”

Other than *S. cerevisiae* and *C. albicans*, Yaf9 proteins from the other fungal species were mined using the HMM model described in (28).

Figure S2

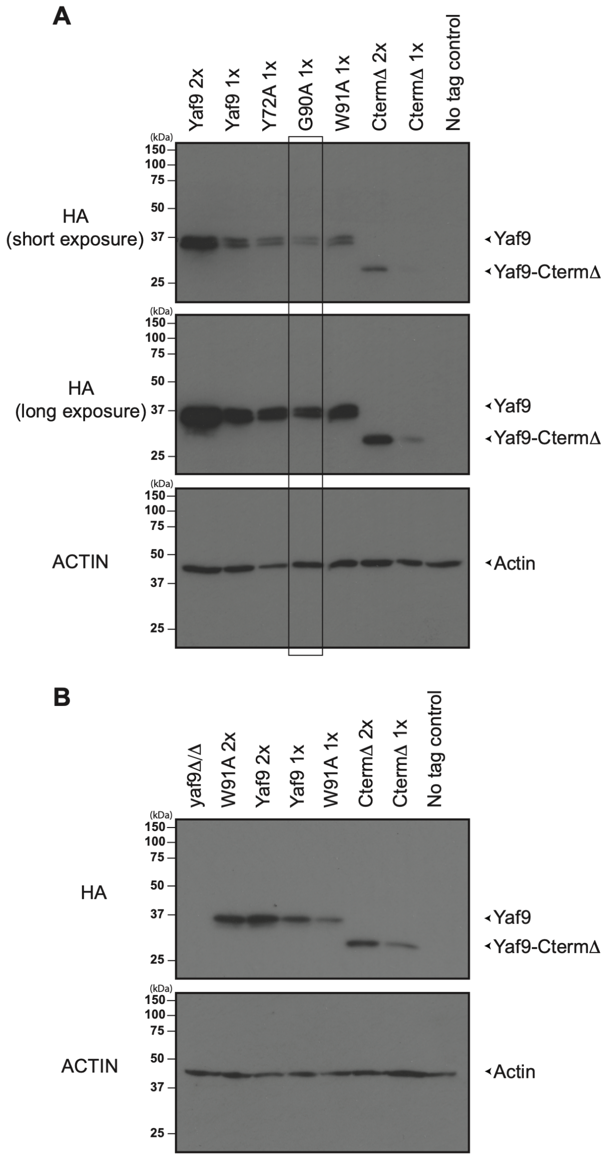

Figure S2. Uncropped western blot images

A. Uncropped western blots for the image shown in Figure 2A. Actin was used a loading control. The rectangle shows the lane that was cropped out in Figure 2A.

B. Uncropped Western blot shown in Figure 2C.

Figure S3

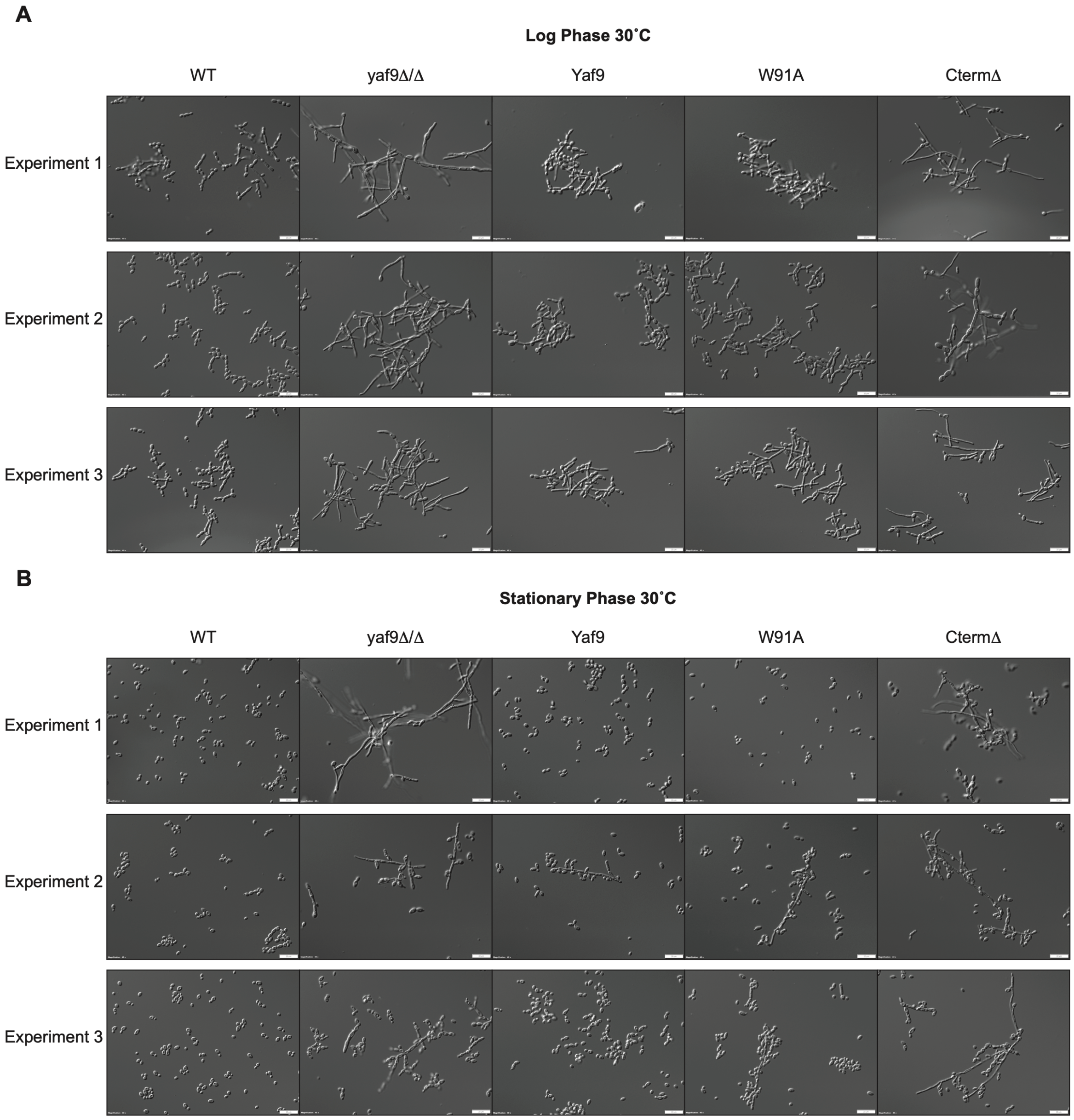

Figure S3. Roles of the Yaf9 YEATS and C-terminal domains in cell morphology of *C. albicans*

- **A.** Morphology images of three independent repeats of indicated strains in log phase growth at 30˚C in YPD + uridine media (3h of growth). Repeat 1 is also shown in Figure 4 (repeated here to have all three repeats for comparison). The scale bar is 20μm.
- **B.** Morphology images of three independent repeats of indicated strains in log phase growth at 30˚C in YPD + uridine media (18h of growth). Repeat 1 is also shown in Figure 4 (repeated here to have all three repeats for comparison). The scale bar is 20μm.

Figure S4

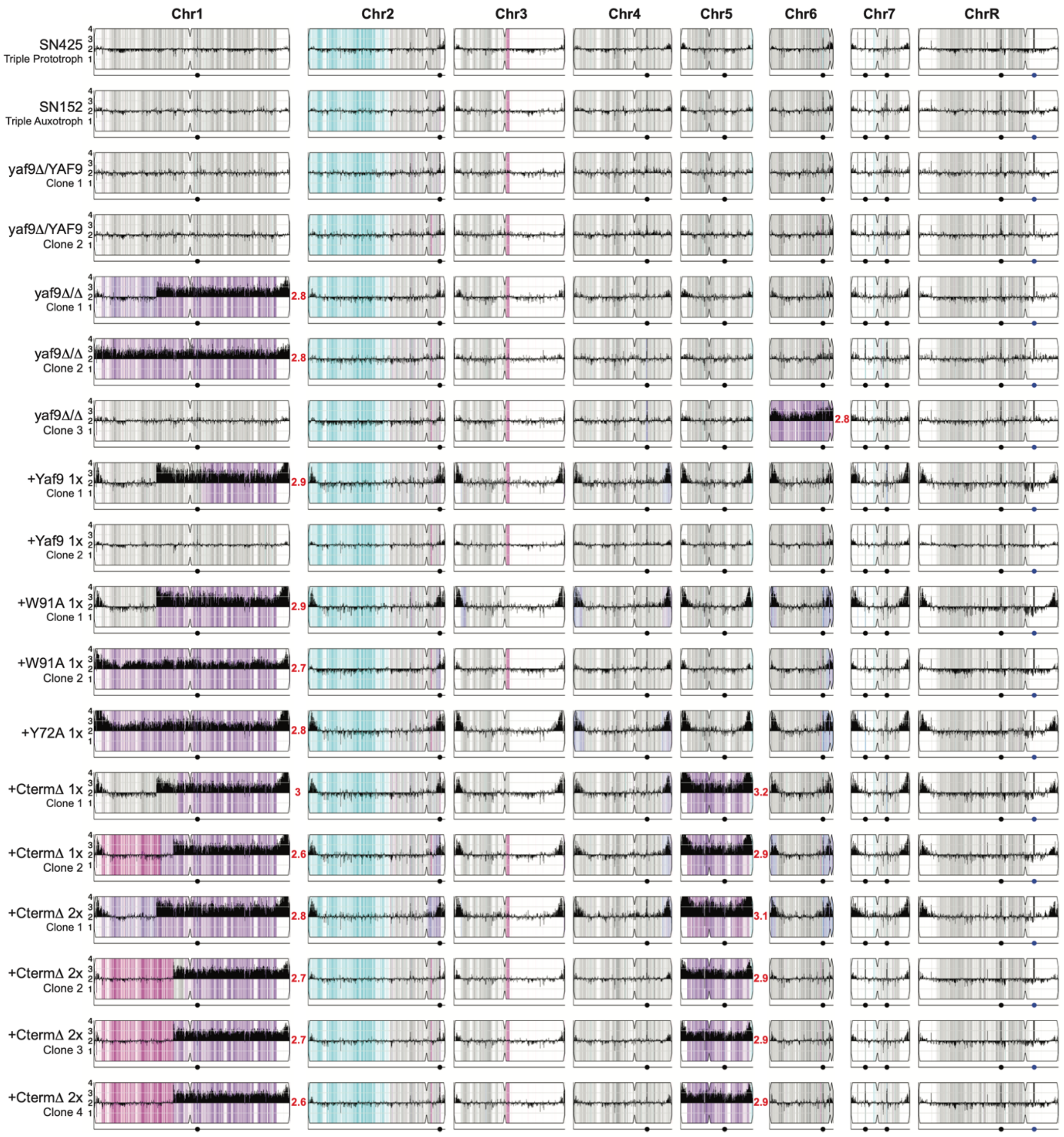

Figure S4. Complex genomic adaptations to loss of *YAF9* in *C. albicans*.

Additional YMAP images of independent clones and its *Yaf9* derivative strains as indicated. Copy number variations per position are displayed as black histograms along the length of each chromosome and copy number calculation is indicated next to each chromosome for those showing whole genome ploidy. A subsection of these strains is shown in Figure 5, and they are repeated here for ease of comparison with the independently generated mutant strains.

Figure S5

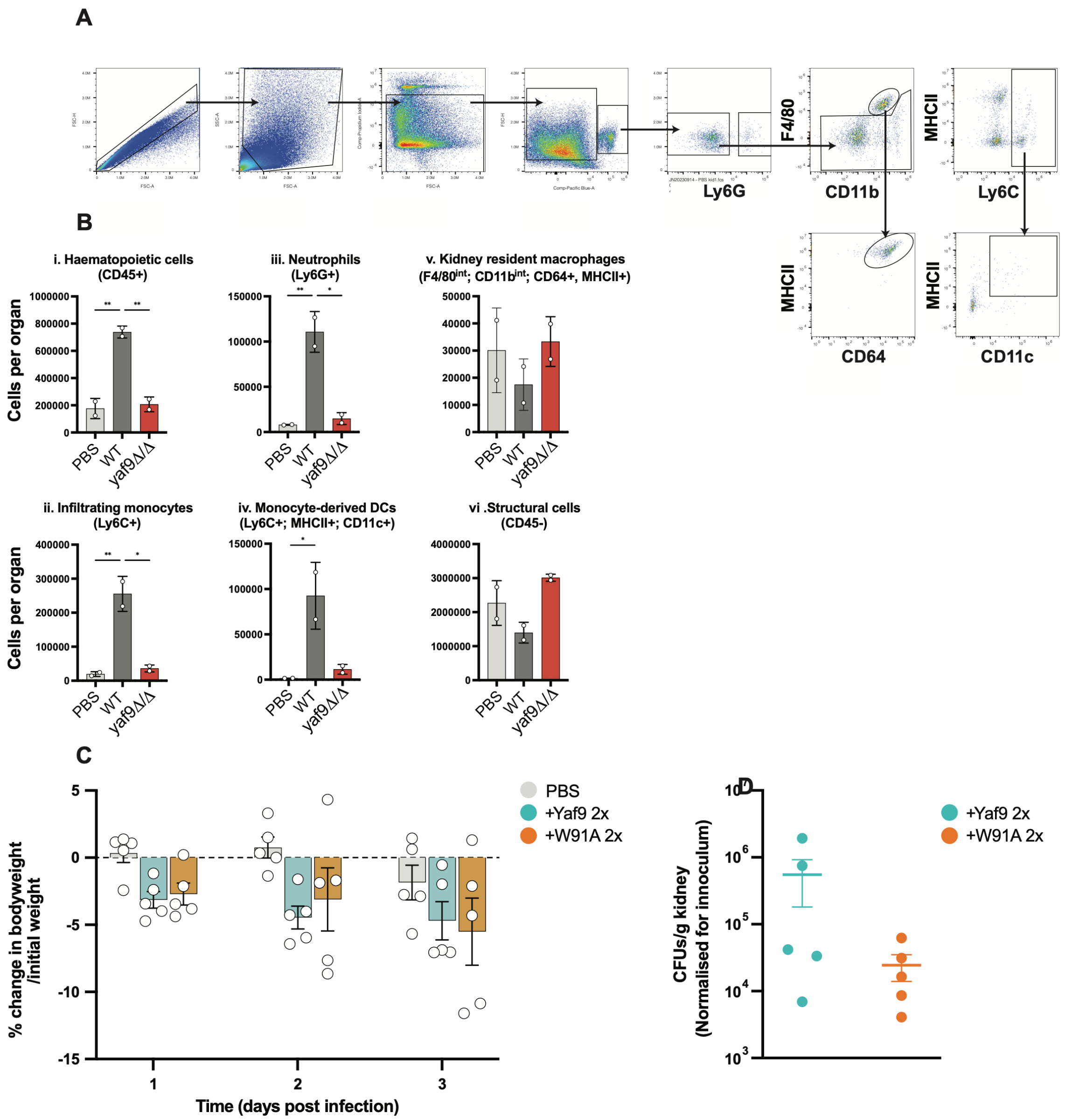
A
Ly6G
F4/80
CD11b
MHCII
Ly6C
MHCII
CD64
MHCII
CD11c
B
i. Haematopoietic cells
(CD45+)
iii. Neutrophils
(Ly6G+)
v. Kidney resident macrophages
(F4/80int; CD11bint; CD64+, MHCII+)
ii. Infiltrating monocytes
(Ly6C+)
iv. Monocyte-derived DCs
(Ly6C+; MHCII+; CD11c+)
vi .Structural cells
(CD45-)
Cells per organ
PBS
WT
yaf9Δ/Δ
C
PBS
+Yaf9 2x
+W91A 2x
% change in bodyweight
/initial weight
Time (days post infection)
D
CFUs/g kidney
(Normalised for innoculum)
+Yaf9 2x
+W91A 2x

Figure S5. Additional analyses of mice infected with the *yaf9* mutant strains

A. Flow Cytometry gating strategy for immune cell enumeration.

B. Kidney immune cell enumeration showing i. Haematopoietic cells (CD45+), ii. Infiltrating Monocytes (Ly6C+), iii. Neutrophils (Ly6G+), iv. Monocyte-derived DCs (Ly6C+, CD11c+, MHCII+ cells), v. Kidney resident macrophages (CD11b+, F4/80+, CD64+ cells) and vi. Kidney structural cells (CD45-). Two mice per strain were analysed. The data points from these two individual animals.

C. Percentage weight change of mice infected with the indicated strains. The "uninfected" mice were mock-infected with sterile 1x PBS. Each data point is from an individual animal. A students T-test statistical analysis was followed by Tukey's multiple comparison, with no statistical significance found. All comparisons are shown in Dataset S1.

D. *C. albicans* colony forming units from kidneys at day 3 post-infection from the experiments in C. Each data point is from an individual animal. CFUs were normalised to the infection inoculum. Shown are the mean and SEM. A students T-test statistical analysis was followed by Tukey's multiple comparison, with no statistical significance found. All comparisons are shown in Dataset S1.
