# Supplementary material for: The C-terminal protein interaction domain of the chromatin reader Yaf9 is critical for pathogenesis of Candida albicans
Source: mSphere. 2024 Feb 20;9(3):e00696-23. doi: 10.1128/msphere.00696-23 (PMC10964406; doi:10.1128/msphere.00696-23)
Supplement: Table S1 — Reagents and strains used in the study. [file msphere.00696-23-s0003.pdf]

Table S1. Reagents and strains

REAGENTS and RESOURCES	SOURCE	IDENTIFIER
Antibodies		
Anti-Actin Antibody, clone C4	Millipore	Cat # MAB1501; RRID:AB_2223041
Anti-HA, 11 Epitope Tag Antibody, Clone16B12	BioLegend	Cat # 901513; RRID:AB_2565335
Anti-Mouse IgG	Sigma-Aldrich	Cat # A4416; RRID:AB_258167
CD45, clone 104, Pacific Blue fluorochrome	Biolegend	Cat # 109820
F4/80, BM8, BV7:11 fluorochrome	Biolegend	Cat # 123147
CD11b, M1-70, APC-Cy7 fluorochrome	Biolegend	Cat # 101226
CD11c, N418, PeCy7 fluorochrome	Biolegend	Cat # 117318
MHCII, 1-A 1-E, BV650 fluorochrome	Biolegend	Cat # 107622
Ly6C+, HK1.4, BV510 fluorochrome	Biolegend	Cat # 128033
Ly6G+, 1A8, BV605 fluorochrome	Biolegend	Cat #127639
CD64, f X54-5/7.1, APC fluorochrome	Biolegend	Cat # 139306
Chemicals		
30% Acrylamide/Bis Solution, 29:1	Biorad	Cat # 161-0156; CAS: 76-06-1,110-26-9
Calcofluor White (CFW)	Sigma-Aldrich	Cat # F3543; CAS: 4404-43-7
Caspofungin (Cancidas)	Merck	Cat # 1583279; CAS: 179463-17-3
CellTracker^{TM} Green CMFDA dye	Thermo Fisher Scientific	Cat # C7025; CAS: 136832-63-8
D- (+)- Glucose	Sigma-Aldrich	Cat # G8270; CAS: 50-99-7
Deoxyribonucleic acid solution from calf thymus	Sigma-Aldrich	Cat # D8661; CAS: 9007-49-2
DRAQ7	Abcam	Cat # AB109202
Fetal Bovine Serum (FBS)	Serana	Cat # FBS-AU-015
Formamide	Sigma-Aldrich	Cat # F9037; CAS: 75-12-7
Hydrogen Peroxide (H_2O_2)	Merck	Cat # 1072090500; CAS: 7722-84-1
Hydroxyurea	Sigma-Aldrich	Cat # H8627; CAS: 127-07-1
Lithium Acetate	Sigma-Aldrich	Cat # L6883; CAS: 546-89-4
Penicillin-Streptomycin	Sigma-Aldrich	Cat # P4333; CAS: 3810-74-0
Peptone	US Biological	Cat # P3302; CAS: 73049-73-7
Phenol:Chloroform:Isoamyl Alcohol 25:24:1	Sigma-Aldrich	Cat # P2069; CAS: 108-95-2, 67-66-3, 123-51-3
Polyethylene glycol (PEG)	Sigma-Aldrich	Cat # P3640; CAS: 25322-68-3
Sodium Chloride (NaCl)	Sigma-Aldrich	Cat # S5886; CAS: 7647-14-5
Sodium Dodecyl Sulfate (SDS)	Sigma-Aldrich	Cat # 75746; CAS: 151-21-3
Sodium Hydroxide	Sigma-Aldrich	Cat # S5881; CAS: 1310-73-2
Trichloroacetic acid (TCA)	Sigma-Aldrich	Cat # T6399; CAS: 76-03-9
Uridine	Sigma-Aldrich	Cat # U3750; CAS: 58-96-8
Yeast Extract	US Biological	Cat # Y2010; CAS: 8013-01-2
Commercial assays		
Clarity Western ECL substrate	Biorad	Cat # 1705060
Wizard® SV Gel and PCR Clean-Up System	Promega	Cat # A9281
Wizard® Plus SV Minipreps DNA Purification System	Promega	Cat # A1460
Mouse and fungal strains		

Mouse: wildtype BALB/c	Monash Animal Research Platform (Melbourne, Australia)	N/A
Mouse: wildtype C57BL/6J	Monash Animal Research Platform (Melbourne, Australia)	RRID: IMSR_JAX:000664
C.albicans: SN152. *leu2Δ/leu2Δ, arg4Δ/arg4Δ, his1Δ/his1Δ, ura3Δ/URA3, iro1Δ/IRO1*	Noble et al., 2010; Wang et al., 2020	YCAT638
C.albicans: SN425. *leu2Δ::C.d.HIS1/leu2Δ::C.m.LEU2, arg4Δ/arg4Δ::C.d.ARG4, his1Δ/his1Δ, ura3Δ/URA3, iro1Δ/IRO1*	Noble et al., 2010; Wang et al., 2020	YCAT641
C.albicans: YAF9/yaf9Δ. YAF9/yaf9Δ::C.m.LEU2, leu2Δ/leu2Δ, arg4Δ/arg4Δ, his1Δ/his1Δ, ura3Δ/URA3, iro1Δ/IRO1	This study	YCAT929, 930
C.albicans: yaf9Δ/Δ. Yaf9Δ::C.d.HIS1/yaf9Δ::C.m.LEU2, leu2Δ/leu2Δ, arg4Δ/arg4Δ, his1Δ/his1Δ, ura3Δ/URA3, iro1Δ/IRO1	This study	YCAT936, 948, 949
C.albicans: yaf9Δ/Δ. Yaf9Δ::C.d.HIS1/yaf9Δ::C.m.LEU2, leu2Δ/leu2Δ::C.d.ARG4, arg4Δ/arg4Δ, his1Δ/his1Δ, ura3Δ/URA3, iro1Δ/IRO1	This study	YCAT972
C.albicans: yaf9Δ/Δ + YAF9 (1X). yaf9Δ::C.d.HIS1/yaf9Δ::C.m.LEU2, leu2Δ/leu2Δ::YAF9-3HA::C.d.ARG4, arg4Δ/arg4Δ, his1Δ/his1Δ, ura3Δ/URA3, iro1Δ/IRO1	This study	YCAT1142, 1248
C.albicans: yaf9Δ/Δ + YAF9 (2X). yaf9Δ::YAF9-3HA::p-A.g.TEF1- C.a.NAT1-t-A.g.TEF1::C.d.HIS1/yaf9Δ::C.m.LEU2, leu2Δ/leu2Δ::YAF9-3HA::C.d.ARG4, arg4Δ/arg4Δ, his1Δ/his1Δ, ura3Δ/URA3, iro1Δ/IRO1	This study	YCAT1175, 1279
C.albicans: yaf9Δ/Δ + yaf9CtermΔ (1X). yaf9Δ::C.d.HIS1/yaf9Δ::C.m.LEU2, leu2Δ/leu2Δ::yaf9CtermΔ-3HA::C.d.ARG4, arg4Δ/arg4Δ, his1Δ/his1Δ, ura3Δ/URA3, iro1Δ/IRO1	This study	YCAT1201, 1251
C.albicans: yaf9Δ/Δ + yaf9W91A (1X). yaf9Δ::C.d.HIS1/yaf9Δ::C.m.LEU2, leu2Δ/leu2Δ::yaf9W91A-3HA::C.d.ARG4, arg4Δ/arg4Δ, his1Δ/his1Δ, ura3Δ/URA3, iro1Δ/IRO1	This study	YCAT1204, 1254
C.albicans: yaf9Δ/Δ + yaf9CtermΔ (2X). yaf9Δ::yaf9CtermΔ-3HA::p-A.g.TEF1- C.a.NAT1-t-A.g.TEF1::C.d.HIS1/yaf9Δ::C.m.LEU2, leu2Δ/leu2Δ::yaf9CtermΔ-3HA::C.d.ARG4, arg4Δ/arg4Δ, his1Δ/his1Δ, ura3Δ/URA3, iro1Δ/IRO1	This study	YCAT1212, 1282, 1283, 1284

C.albicans: yaf9Δ/Δ + yaf9W91A (2X). yaf9Δ::yaf9W91A-3HA::p-A.g.TEF1- C.a.NAT1-t-A.g.TEF1::C.d.HIS1/yaf9Δ::C.m.LEU2, leu2Δ/leu2Δ::yaf9W91A-3HA::C.d.ARG4, arg4Δ/arg4Δ, his1Δ/his1Δ, ura3Δ/URA3, iro1Δ/IRO1	This study	YCAT1215
C.albicans: yaf9Δ/Δ + yaf9Y72A (1X). yaf9Δ::C.d.HIS1/yaf9Δ::C.m.LEU2, leu2Δ/leu2Δ::yaf9Y72A-3HA::C.d.ARG4, arg4Δ/arg4Δ, his1Δ/his1Δ, ura3Δ/URA3, iro1Δ/IRO1	This study	YCAT1257
Others		
10mm nylon filter	Millipore	Cat # NY1004700
Software		
CellProfiler 2.1.1	Kamentsky et al., 2011	N/A
ChimeraX	RBVI, CGL, NIH	https://www.rbvi.ucsf.edu/chimerax/
ImageJ 2.0.0-rc-69	NIH	https://imagej.nih.gov/ij/download.html
GraphPad Prism 9.0.0	GraphPad Software, San Diego, California USA	https://www.graphpad.com/scientific-software/prism/
Growthcurver package	Rstudio, PBC, Boston, MA, USA	https://cran.rproject.org/web/packages/growthcurver/vignettes/Growthcurvervignette.html#output-metrics
Yeast Mapping Analysis Pipeline (YMAP)	Abbey et al., 2014	http://lovelace.cs.umn.edu/Ymap/
