# Supplementary material for: The C-terminal protein interaction domain of the chromatin reader Yaf9 is critical for pathogenesis of Candida albicans
Source: mSphere. 2024 Feb 20;9(3):e00696-23. doi: 10.1128/msphere.00696-23 (PMC10964406; doi:10.1128/msphere.00696-23)
Supplement: Table S2 — Primers used in the study. [file msphere.00696-23-s0004.pdf]

Table S2. List of primers

Name	Purpose	5' to 3' sequence	Source
Candida genes			
**CAT734 F**	For checking 3' end of C.m. LEU2 marker	GCAATATCTTTCAATGCCATT	This study
**CAT735 R**	For checking 5' end of C.m. LEU2 marker	GGTATCAGAACTGCAGATTTAAG	This study
**CAT737 R**	For checking 3' end of C.d. HIS1 marker	CACCTTTAAAATCAATGGGC	This study
**CAT747 R**	For checking 5' end of C.m. LEU2 marker	GGTATCAGAACTGCAGATTTAAG	This study
**CAT752 F**	For checking integration of 3' end of C.d. ARG4 marker in LEU2 locus	CCATTGTGAAGATCGTCTGA	This study
**CAT758 R**	For checking integration of C.d. ARG4 marker in LEU2 locus; *LEU2* specific primer	GATCGAACAACAATAACTACC	This study
**CAT759 F**	For checking integration of C.d. ARG4 marker in LEU2 locus; *LEU2* specific primer	ATTAATGAAGCAAGAATCGC	This study
**CAT855 F**	For checking 5' end of C.d. HIS1 marker	CGTATAATAATTCTAGTATG	This study
**CAT964 F**	For checking *YAF9* upstream	TAACAAGCCTTTCCCCCTTT	Wang et al., 2020
**CAT965 R**	For checking *YAF9* ORF	GGTGTTGTTGGTTTCCGTTT	This study
**CAT966 R**	For checking *YAF9* downstream	CAAGGTTGTTGGAGGGATGT	Wang et al., 2020
**CAT968 F**	For checking *YAF9* internal ORF	ACCAACAACACCACCAGAACA	Wang et al., 2020
**CAT969 R**	For checking *YAF9* internal ORF	TTGTTGCGGTGGTTGAGCTT	Wang et al., 2020
**CAT1075 F**	For checking *YAF9* upstream	CAACAACCCACTTGTTCTTT	This study
**CAT1076 R**	For checking 5' end of *YAF9* ORF	AAATTGTTGTTGCGGTGGTT	This study
**CAT1077 F**	For checking 3' end of *YAF9* ORF	GGAGAAGTTCATCTGGTATTATATG	This study
**CAT1078 F**	For checking *YAF9* at 3HA tag end	AGAAAAAGCTTCATGGCCTT	This study
**CAT1079 F**	For checking *YAF9* second copy	GGTTAGGATTTGCCACTGAG	This study
**CAT1080 F**	For checking 3' end of NAT1 marker	TGAATTCGCCAGAGAAAGAG	This study
**CAT1081 R**	For checking the integration of 3HA tag at 3'	CGACCGAGATTCCCGGGTAA	This study
**CAT1082 F**	For checking *YAF9* ORF	CCTTTCTCCATTAACAGTAATAGTTTG	This study
**CAT1083 R**	For checking 5' end of C.d. HIS1 marker	GCGAATTCTGCAGATATCCA	This study
**CAT1084 F**	For checking *YAF9* internal ORF	ATCAAGTCAC AGAAACTGGA	This study
**CAT1085 R**	For checking *YAF9* internal ORF	TTCATCAATTTCATCGGGTC	This study
**CAT1086 F**	For checking integration of C.d. ARG4 marker in LEU2 locus; *LEU2* specific primer	TGGGAAATGG TTCAAGAAGG	This study
**CAT1087 R**	For checking integration of C.d. ARG4 marker in LEU2 locus; *LEU2* specific primer	AATGAAGAGCCAAATCATATACG	This study
**CAT1088 R**	For checking 5' end of NAT1 marker	CTCAGTGGCAAATCCTAACC	This study
**CAT1089 R**	For checking integration of 5' end C.d. ARG4 marker	TGACGTAATTCCTATCTCTCTT	This study
**CAT1156 F**	For checking internal C.m. LEU2 marker	GTAAAGCCATGAAGGCAGCC	This study

**CAT1157 R**	For checking internal C.m. LEU2 marker	CATCATTTAATCGGTGGTGCTGC	This study
**CAT1158 F**	For checking internal C.d. HIS1 marker	CCAGCAGCTTTCATGGTCT	This study
**CAT1159 R**	For checking internal C.d. HIS1 marker	GAGCGGTGCCGATATACAG	This study
**CAT1160 R**	For checking *YAF9* second copy containing NAT1 marker	GTAGCCAAACCCATCAAAGC	This study
**CAT1161 R**	For checking NAT1 marker	TCAACGCGTCTGTGAGG TTTCGCCTCGACATCATCTGC	This study
**CAT1162 F**	For checking NAT1 marker	TTTCGCCTCGACATCATCTGC	This study
**CAT1321 R**	For checking *YAF9* upstream	GATGACATGGATTAAGGTGGGAG	This study
**CAT1322 F**	For checking *YAF9* ORF	AACTCATTCAAGAAGAATAAAATTTG TATCG	This study

F = Forward primer
R = Reverse primer
ORF = Open reading frame
C.d. = *Candida dublinensis*
C.m. = *Candida maltosa*
